# A Multi-Exon-Skipping Detection Assay Reveals Surprising Diversity of Splice Isoforms of Spinal Muscular Atrophy Genes

**DOI:** 10.1371/journal.pone.0049595

**Published:** 2012-11-19

**Authors:** Natalia N. Singh, Joonbae Seo, Sarah J. Rahn, Ravindra N. Singh

**Affiliations:** Department of Biomedical Sciences, Iowa State University, Ames, Iowa, United States of America; Oregon Health & Science University, United States of America

## Abstract

Humans have two near identical copies of *Survival Motor Neuron* gene: *SMN1* and *SMN2*. Loss of *SMN1* coupled with the predominant skipping of *SMN2* exon 7 causes spinal muscular atrophy (SMA), a neurodegenerative disease. SMA patient cells devoid of *SMN1* provide a powerful system to examine splicing pattern of various *SMN2* exons. Until now, similar system to examine splicing of *SMN1* exons was unavailable. We have recently screened several patient cell lines derived from various diseases, including SMA, Alzheimer’s disease, Parkinson’s disease and Batten disease. Here we report a Batten disease cell line that lacks functional *SMN2*, as an ideal system to examine pre-mRNA splicing of *SMN1*. We employ a multiple-exon-skipping detection assay (MESDA) to capture simultaneously skipping of multiple exons. Our results show surprising diversity of splice isoforms and reveal novel splicing events that include skipping of exon 4 and co-skipping of three adjacent exons of *SMN*. Contrary to the general belief, MESDA captured oxidative-stress induced skipping of *SMN1* exon 5 in several cell types, including non-neuronal cells. We further demonstrate that the predominant *SMN2* exon 7 skipping induced by oxidative stress is modulated by a combinatorial control that includes promoter sequence, endogenous context, and the weak splice sites. We also show that an 8-mer antisense oligonucleotide blocking a recently described GC-rich sequence prevents *SMN2* exon 7 skipping under the conditions of oxidative stress. Our findings bring new insight into splicing regulation of an essential housekeeping gene linked to neurodegeneration and infant mortality.

## Introduction

Alternative pre-mRNA splicing increases the coding potential of eukaryotic genome by producing multiple proteins from a single gene [1]. As per current estimate, more than 95% of human genes with two or more exons are alternatively spliced [2]. Splicing is catalyzed by spliceosome, a macromolecular machine, which is assembled de novo for the removal of each intron [3], [4]. Splicing is also coupled with transcription as several splicing factors are recruited to spliceosome and/or pre-mRNA sequence through RNA polymerase [5]. Regulation of alternative splicing rests on non-spliceosomal factors that bind to pre-mRNA sequences called exonic or intronic splicing enhancers (ESEs or ISEs) and silencers (ESSs or ISSs) [6]–[8]. Enhancer and silencer motifs promote or suppress splice-site (ss) selection, respectively. Due to the difference in arrangement of cis-elements within exonic and flanking intronic sequences, regulation of alternative splicing of each exon is unique [9], [10]. Mutations within regulatory sequences and/or aberrant expression of splicing factors due to genotoxic and/or oxidative stress (OS) result in defective splicing [11]–[17]. However, there are very limited studies capturing OS-triggered aberrant splicing of multiple exons in a single transcript of an essential human gene. Also it is not known if deleterious effect of OS on splicing of a specific exon could be prevented by strengthening of a ss.

**Figure 1:**
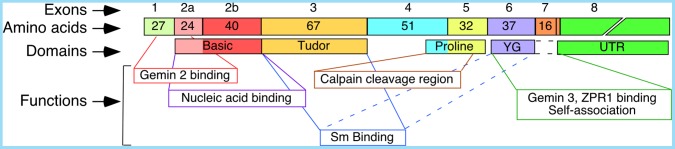
Diagrammatic representation of *SMN* mRNA and SMN protein. Number of amino acids encoded by each exon is indicated. Exons as well as domains they encode are shown as boxes. Domain functions are indicated.

Humans have two near identical copies of *Survival Motor Neuron* gene: *SMN1* and *SMN2* [18]. Hereafter, *SMN* (in italics) refers to a gene or a transcript, whereas SMN (in normal case capital letters) refers to a protein. Both *SMN* genes code for identical proteins; however, *SMN2* predominantly generates a short transcript due to skipping of exon 7, producing a truncated protein (SMN*Δ*7) that is highly unstable [19]–[21]. Therefore, *SMN1* serves as the primary gene for production of full-length SMN, a multifunctional protein containing nucleic acid binding, tudor, Sm binding, Calpain cleavage, ZPR1 binding and Gemin2 binding domains (Figure 1) [22]–[28]. Interaction of SMN with Gemin2 is essential for the formation of a large heteromeric complex (SMN complex) that participates in snRNP biogenesis, an important housekeeping function [29], [30]. SMN is also implicated in transcription, DNA recombination, signal transduction, stress granule formation, vesicular transport and motor neuron trafficking [31]–[37]. The inability of *SMN2* to compensate for the loss of *SMN1* results in spinal muscular atrophy (SMA), a leading genetic cause of infant mortality [38]. The exact function of *SMN2* remains unknown, although, several lines of evidence support its role in cellular metabolism. For instance, a SMA mouse model expressing very high levels of SMN*Δ*7 showed prolonged lifespan [39]. Further, deletion of *SMN2* has been associated with higher incidence of amyotrophic lateral sclerosis (ALS) and lower motor neuron disease [40], [41]. In addition, *SMN2* serves as a spare gene with a potential to be corrected in SMA. Indeed, recent reports of correction of *SMN2* exon 7 splicing in animal models have shown promise for SMA therapy [42]–[47]. Most lead compounds to show therapeutic potential in animal models have been initially found to correct *SMN2* exon 7 splicing in cultured SMA patient cells. In particular, publically available GM03813 cell line that lacks *SMN1* has emerged as a cell-based model system for the preliminary screening of potential SMA drugs [48]–[54]. GM03813 cell line has also been useful in validating regulatory cis-elements and transacting factors that modulate *SMN2* exon 7 splicing [48], [51]–[55]. On the other hand, there is no systematic study on *SMN1* splicing regulation in a publically available *SMN2*-lacking cell line.

**Figure 2:**
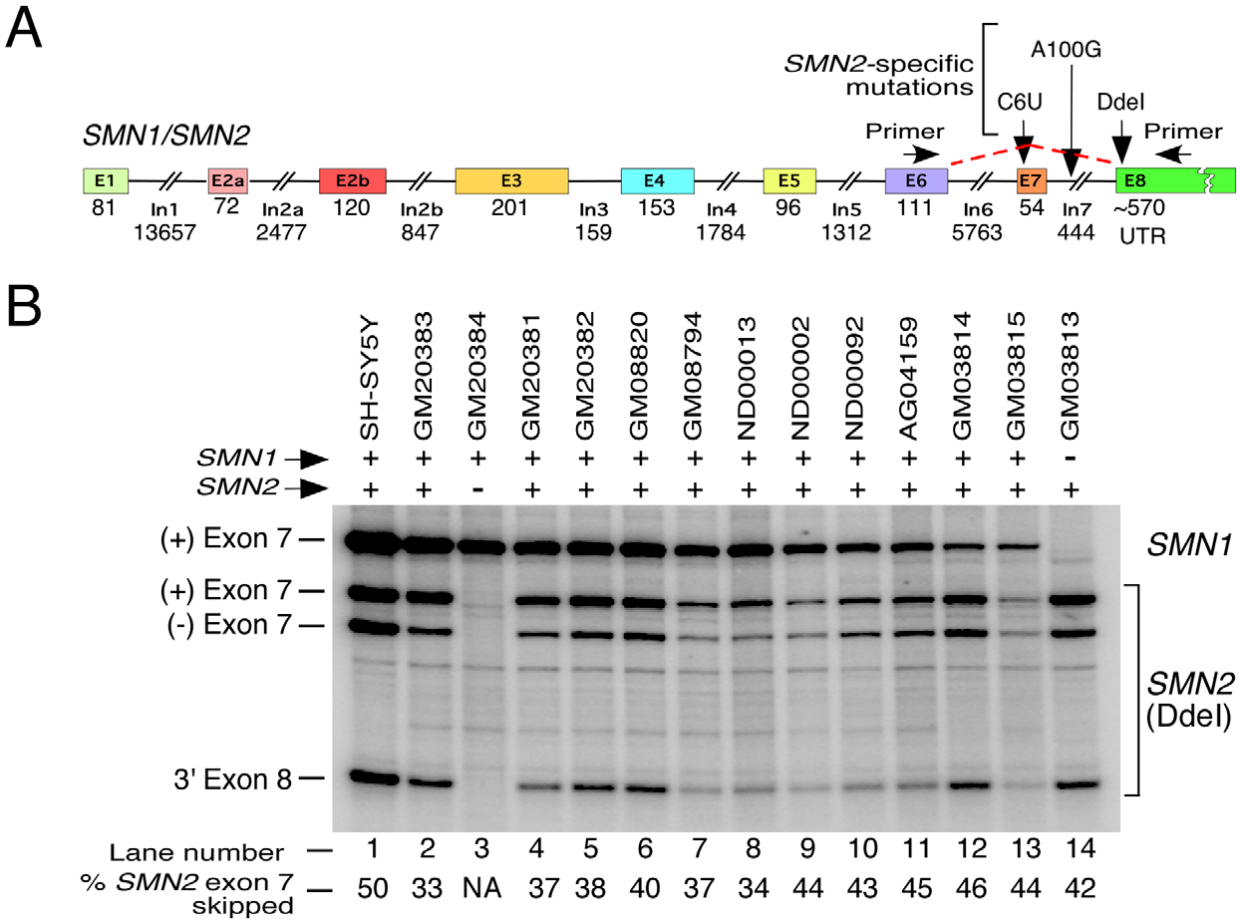
Splicing of endogenous *SMN* exon 7. *A,* Diagrammatic representation of exon/intron organization of *SMN1* and *SMN2* genes. Exonic sequences are shown as colored boxes and intronic sequences are shown as lines/broken lines. Sizes of exons and introns are given. Size of the 3′UTR is based on the prevalently reported *SMN1* transcript (NCBI accession number NM_000344). ***B,*** Splicing patterns of endogenous *SMN1* and *SMN2* exon 7 in different cell lines. Name of cell lines used are given on the top of the gel. Spliced products were analyzed by RT-PCR. We used 1 µg of total RNA per 5 µl of reverse transcription reaction and generated cDNA employing random primers. *SMN* transcripts were amplified using primers P25 and P31. To distinguish transcripts originated from *SMN2*, PCR products were digested with DdeI [55]. *SMN1* and *SMN2* transcripts are indicted on the right of the gel. Exon 7-included (+) and exon 7-skipped (−) products are indicated on the left of the gel. Based on RT-PCR results, the presence and absence of *SMN1/SMN2* gene is marked by (+) and (−) respectively. The percentage of *SMN2* exon 7 skipping was calculated based on the total value of *SMN2* exon 7-included and exon 7-skipped products [55].The last 81 nucleotides of exon 1 are translated. Several mutations that distinguish *SMN1* and *SMN2*, including DdeI restriction site, as well as location of primers used for amplification of endogenous *SMN* transcripts are indicated.

Both *SMN1* and *SMN2* have similar gene organization i.e. nine exons and eight introns (Figure 2A). A critical cytosine (C) to thymidine (T) mutation at the 6^th^ position (C6U transition in transcript) of exon 7 and an adenosine (A) to guanosine (G) transition at the 100^th^ position (A100G) of intron 7 cause *SMN2* exon 7 skipping [19], [56]. Both, C6U and A100G mutations create binding sites for an inhibitory protein hnRNP A1 that weakens the 3′ ss of *SMN2* exon 7 [56]. An additional G to A mutation at the 236^th^ position (G236A) of non-coding exon 8 creates a *SMN2*-specific signature motif (CTNAG) that could be cleaved by DdeI restriction endonuclease (Figure 2A). Therefore, DdeI digestion has been useful in distinguishing *SMN2* transcripts from *SMN1* transcripts [19]. Based on studies in SMA patient cells as well as in mouse models carrying *SMN2*, skipping of *SMN2* exons 3, 5 and 7 have been confirmed [18], [48], [53], [57]. There is also evidence to suggest very small but detectable skipping of *SMN1* exon 5 and exon 7 in certain cell types [18], [19], [58]. However, it is not known if splicing of two or more exons of *SMN* is co-regulated. Also, there is no report of skipping of *SMN1* exon 3. In general, there is a lack of a reliable assay to capture the relative abundance of the major splice variants of *SMN1* and *SMN2*.

Paraquat (PQ), an herbicide and oxidative stress (OS)-causing agent, has been linked to the increased risks of neurological disorders, including Parkinson’s disease [59]. Incidentally, PQ treatment of neuronal cells have been shown to cause enhanced skipping of exons 5 and 7 of *SMN2* but not *SMN1* [60]. Although not independently validated, these findings put SMA patients into the category of high-risk candidates who are likely to be affected the most under the conditions of OS. In addition, OS-induced enhanced skipping of *SMN2* exon 7 raises the fundamental question whether any of the strategies aimed at the correction of *SMN2* exon 7 splicing in SMA will retain its efficacy under the conditions of OS. Given a high degree of sequence conservation between *SMN1* and *SMN2*, it is likely that some of the yet uncharacterized splicing events in OS are common to both, *SMN1* and *SMN2*. A definitive answer to this question awaits further experimentation in specific cell types that express either *SMN1* or *SMN2*.

Here we report a systematic analysis of splice isoforms generated by *SMN1* and *SMN2* under normal and OS conditions. Our study also addresses an important question of neuronal versus non-neuronal regulation of alternative splicing of various *SMN* exons. One of the defining aspects of this study is the application of a multiple-exon-skipping detection assay (MESDA) that captures the relative abundance of all major splice variants of *SMN*. We also take advantage of a unique cell type (GM20384) that we serendipitously discovered to lack *SMN2* transcripts probably due to a partial or complete deletion of *SMN2* alleles. Our findings reveal novel splice variants, including those generated by an unexpected skipping of exon 4 and/or several adjacent exons of *SMN*. We show that OS affects alternative splicing of several exons of *SMN1* and *SMN2* in both, neuronal and non-neuronal cells. Our results suggest an OS-induced collaborative skipping of *SMN2* exons 5 and 7. Further, we demonstrate that skipping of *SMN2* exon 7 under OS is dependent upon several factors including promoter sequence, endogenous context and the strength of ss. We also demonstrate that an antisense oligonucleotide (ASO)-based strategy to correct *SMN2* exon 7 splicing retains its efficacy under the conditions of OS.

## Results

### Identification of a Cell Line that Lacks *SMN2*

To explore the possibility that different pathological conditions can affect splicing of *SMN* exon 7, we screened a number of publically available patient cell lines, including batten disease (BD), Parkinson’s disease and Alzheimer’s disease cell lines (Table 1, Figure 2B). As a control, we also used undifferentiated neuronal SH-SY5Y cells (lane 1, Figure 2B). *SMN1*/*SMN2* transcripts were analyzed by a sensitive radioactive RT-PCR using forward and reverse primers annealing to exon 6 and exon 8, respectively. Importantly, PCR products contained the *SMN2*-specific DdeI restriction site within exon 8 (Figure 2A). Therefore, after DdeI digestion of PCR products, samples from cells containing both, *SMN1* and *SMN2*, produced four bands (Figure 2B). The slowest migrating band (top band) in a polyacrylamide gel represented the exon 7 included transcripts from *SMN1*, whereas, three fast migrating bands originated from *SMN2*.

**Table 1:** Cell lines used in this study.

No.	Cell type	Source	Disease	Description	Cell Type
1	SH-SY5Y (CRL-2266)	ATCC	NB	Unique marker onChromosome 1	Neuroblastoma
2	GM 20383	CCR	BD/CL	Juvenile CL, CLN3 mutations	B-Lymphocyte
3	GM 20384	CCR	BD/CL	Juvenile CL, CLN3 mutations	B-Lymphocyte
4	GM 20381	CCR	BD/CL	Juvenile CL, CLN3 mutations	B-Lymphocyte
5	GM 20382	CCR	BD/CL	Juvenile CL, CLN3 mutations	B-Lymphocyte
6	GM 08820	CCR	BD/CL	Juvenile CL, CLN3 mutations	B-Lymphocyte
7	GM 08794	CCR	BD/CL	Juvenile CL, CLN3 mutations	B-Lymphocyte
8	ND 00013	CCR	PD	Late onset DNA panel	B-Lymphocyte
9	ND 00002	CCR	PD	Young onset DNA panel	B-Lymphocyte
10	ND 00092	CCR	Ctrl PD	Convenience control	B-Lymphocyte
11	AG 04159	CCR	AD	Familial, type 3	Fibroblast
12	GM 03814	CCR	SMA Carr	Mother (*SMN1+/−*)	Fibroblast
13	GM 03815	CCR	SMA Carr	Father (*SMN1+/−*)	Fibroblast
14	GM 03813	CCR	SMA	Child (*SMN1−/−*), SMA Type I	Fibroblast

**Abbreviations:** ATCC, American type cell collection; CCR, Coriell cell repositories; NB, Neuroblastoma; BD, Batten disease; CL, Ceroid lipofuscinosis; PD, Parkinson’s disease; Ctrl, Control; AD, Alzheimer’s disease; SMA, Spinal muscular atrophy; Carr, Carrier.

Despite similar amount of starting material (RNA) used for RT-PCR, we observed varied intensity of expected four bands in different cell types. This could be due to a combination of factors, including but not limited to variations in *SMN1*/*SMN2* copy numbers, transcription rate and splicing regulation. In order to compare side-by-side the relative proportions of *SMN1* versus *SMN2* transcripts in various cell types, loading of PCR products in polyacrylamide gel was adjusted (Figure 2B). Our assay reliably detected the presence and/or absence of major transcripts specific to *SMN1* and/or *SMN2*. For instance, the *SMN1*-associated top band was absent in GM03813, a well-studied SMA type I patient fibroblast cell line (Figure 2B, lane 14). Except GM03813, all other cell lines in our screening showed the presence of *SMN1*. Noticeably, GM20384, a BD patient lymphocyte cell line, lacked all bands corresponding to *SMN2*, suggesting a complete or partial deletion of both *SMN2* alleles (Figure 2B, lane 3). None of the other five BD patient lymphocytes showed the loss of *SMN2* transcripts. To further ascertain that all exon 7-included products in GM20384 originated from *SMN1*, we sequenced ten clones derived from the top band (full-length transcript). All clones lacked *SMN2* associated signature mutations in exons 7 and 8, confirming the absence of the intact *SMN2* gene. Of note, donor of GM20384 had a mutation in *CLN3* gene that is generally associated with BD (Table 1) [61]. However, irrespective of the presence or absence of *SMN2*, CLN3 mutations did not produce any change in splicing pattern of *SMN1* in any of the BD patient cell lines we tested (Figure 2B). In addition, splicing pattern of *SMN1* in GM20384 cells was similar to those in non-BD cell types. We believe that GM20384 cells provide a valuable tool to understand *SMN1*-specific splicing regulation.

### A Unique Assay Captures Relative Expression of Major Splice Isoforms of *SMN1* and *SMN2*

To determine the relative abundance of major splice variants of *SMN1*/*SMN2* in a one-step reaction, we developed a PCR-based assay that we named “MESDA”. The 5′ and 3′ primers used in MESDA annealed to *SMN* exons 2b and 8, respectively. Our rationale to use 5′ primer in exon 2b was based on the fact that this exon is constitutively spliced. Also, use of 5′ primer in exon 2b versus constitutively spliced exon 1 or exon 2a resulted in shorter PCR products that can be better resolved on a polyacrylamide gel. To maintain the sensitivity and an accurate estimate of the relative molecular abundance of amplified products of different sizes, we performed a limited-cycle radioactive PCR in which only one of the primers was 5′-radiolabeled. Considering that the 5′ and 3′ primers employed in MESDA annealed to exons 2B and 8 of *SMN*, respectively, we were able to capture multiple splice variants, including the transcripts generated by simultaneous skipping of three exons of *SMN*. MESDA also identified novel splice variants due to an entirely unexpected splice site pairings. Cloning and sequencing confirmed identity of splice variants generated by MESDA. GenBank accession numbers of novel splice isoforms of *SMN* reported here are given in Table 2.

**Table 2:** Description and GenBank accession numbers of splice variants reported in this study.

Exon	Gene	Cell line	Treatment*	GenBank accession number
Δ5	*SMN1*	GM20384	PQ-treated	JQ657798
Δ3	*SMN1*	GM20384	PQ-treated	JQ657800
Δ5,7	*SMN1*	GM20384	PQ-treated	JQ657799
Δ5,6,7	*SMN1*	GM20384	PQ-treated	JQ657801
Δ5,6	*SMN1*	GM20383	Untreated	JQ732166
Δ5,6	*SMN2*	GM20383	Untreated	JQ732167
Δ5	*SMN1*	SH-SY5Y	PQ-treated	JQ657802
Δ3	*SMN2*	SH-SY5Y	PQ-treated	JQ690861
Δ3,4	*SMN1*	SH-SY5Y	PQ-treated	JQ745297
Δ4,7	*SMN2*	SH-SY5Y	PQ-treated	JQ690864
Δ3,7	*SMN2*	SH-SY5Y	PQ-treated	JQ690862
Δ3,5	*SMN1*	SH-SY5Y	PQ-treated	JQ657803
Δ3,5,7	*SMN2*	SH-SY5Y	PQ-treated	JQ690863
Δ5,7	*SMN2*	SH-SY5Y	Untreated	JQ690865
Δ3,7	*SMN1*	SH-SY5Y	Untreated	JQ690867
Δ3,5	*SMN2*	SH-SY5Y	Untreated	JQ690866
Δ3,5,7	*SMN1*	SH-SY5Y	Untreated	JQ690868

* Represents whether splice isoforms were detected in untreated and/or PQ-treated cells. PQ-treated refers to treatment of cells with 1 mM PQ for 24 h.

**Figure 3:**
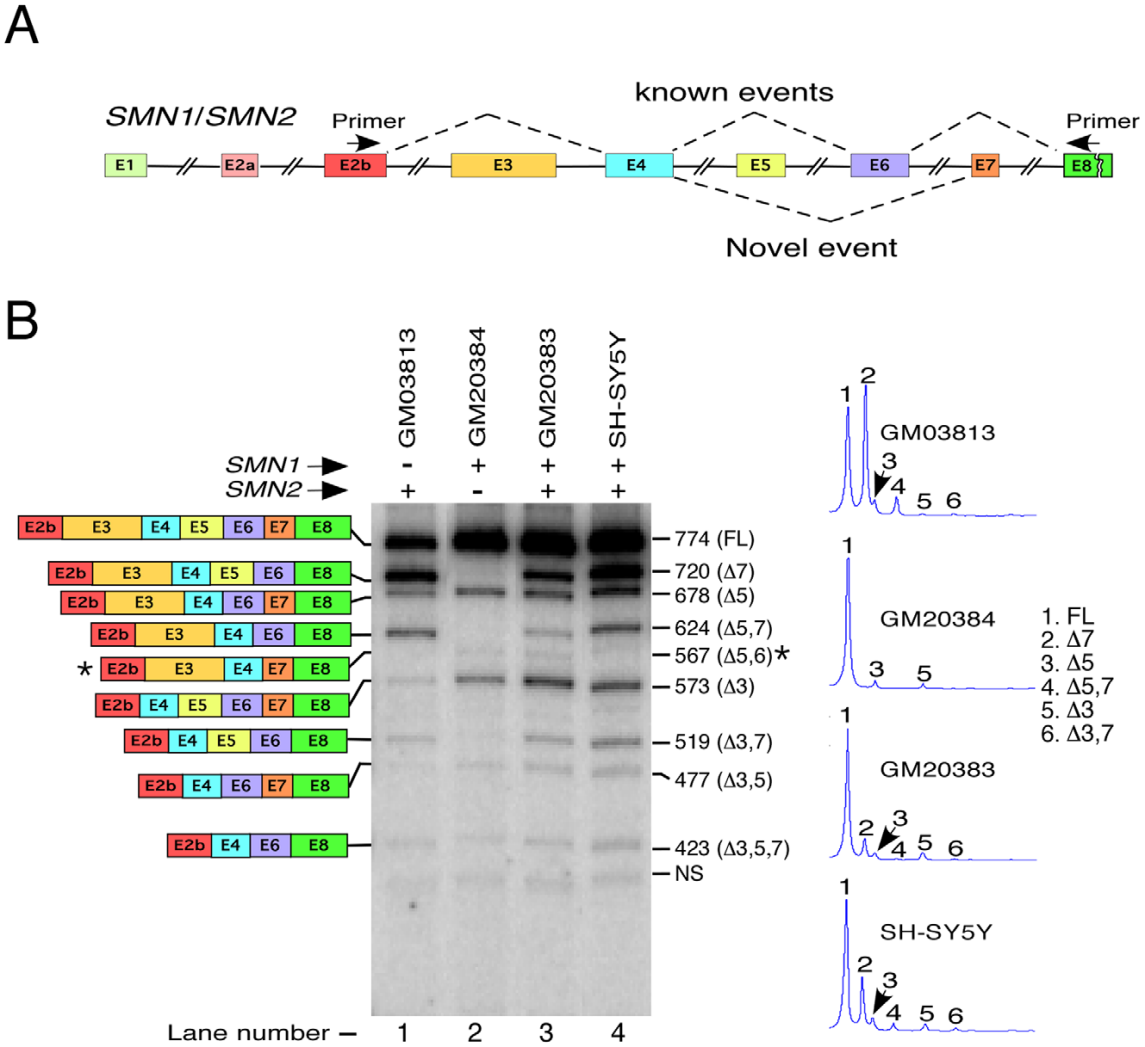
Detection of multiple exon skipping events in endogenous *SMN*. *A,* Diagrammatic representation of the *SMN* gene. Exonic and intronic sequences are depicted as in (Figure 2A). Dotted lines indicate skipping of previously reported exons and/or novel splice variants. Annealing positions of primers used for MESDA are shown. ***B,*** Multiple splice isoforms of endogenous *SMN* pre-mRNA in different cell lines. Name of cell lines used are given on the top of the gel. Splice products were analyzed by RT-PCR. We used 1.4 µg of total RNA per 10 µl of reverse transcription reaction and generated cDNA employing oligo(dT)_12–18_ primer. *SMN* transcripts were amplified using primers 5′hSMN-E2b (anneals within Exon 2b) and P2–2 (anneals within Exon 8). Diagrammatic representation of splice variants is given on the left of the gel; their sizes are indicated on the right of the gel with names of skipped exons given in brackets. For intra-lane comparison of bands, pictograms (shown on right) were generated for each lane by MultiGauge software version 3.0 (FUJIFILM). The identity of splice variants was established by sequencing. Novel splice variant is marked by a star. Note that 567-nucleotide-long Δ5,6 variant moves slower than 573-nucleotide-long Δ3 variant. FL, full length; NS, non-specific. The number of *SMN1* and *SMN2* transcripts for each of splice variants, except for the full-length and Δ7, was determined by sequencing of at least 8 randomly selected clones per variant (Figure S1).

**Figure 4:**
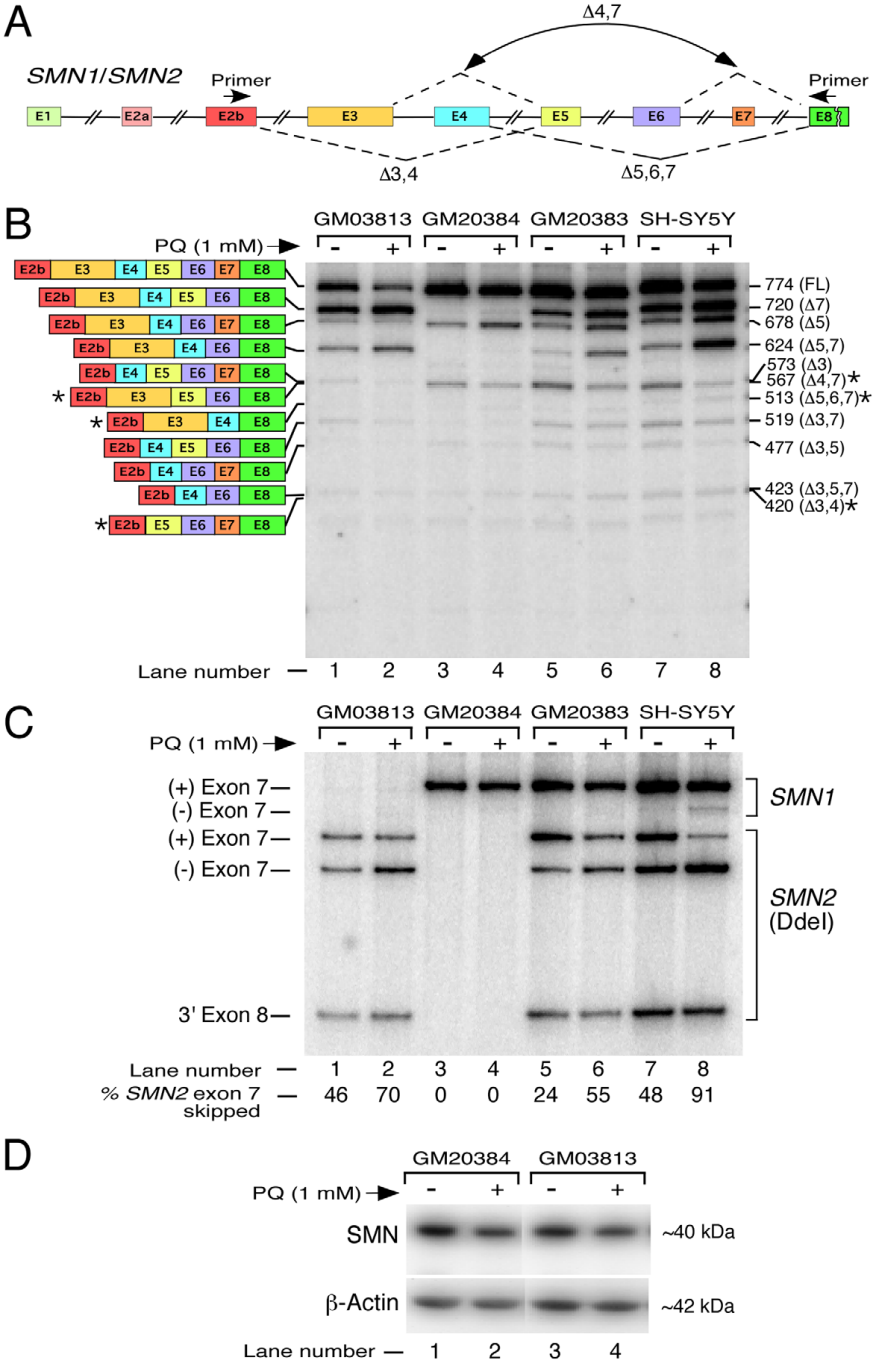
Effect of PQ treatment on splicing of *SMN*. *A,* Diagrammatic representation of the *SMN* gene. Exonic and intronic sequences are depicted as in (Figure 2A). Dotted lines indicate exon skipping that generated novel splice variants. Annealing positions of primers used for MESDA are shown. ***B,*** Detection of multiple exon skipping events in endogenous *SMN* in the presence (+) and absence (−) of PQ. Names of cell lines used are given on the top of the gel. Total RNA was prepared from cells treated with 1 mM of PQ for 24 hours. Splice products were analyzed by RT-PCR as described in Figure 3B. Diagrammatic representation of splice variants is given on the left of the gel; their sizes are indicated on the right of the gel with names of skipped exons given in brackets. The identity of splice variants was confirmed by sequencing. Note that 513-nucleotide-long Δ5,6,7 variant moves slower than 519-nucleotide-long Δ3,7 variant. Novel splice variants are marked by stars. FL, full length; NS, non-specific. For intra-lane comparison of bands, pictograms were generated for each lane (Figure S2). ***C,*** Splicing patterns of endogenous *SMN* exon 7 in the presence (+) or absence (−) of PQ. Names of cell lines used are given on the top of the gel. Total RNA was prepared from cells treated with 1 mM of PQ for 24 hours. Spliced products were analyzed by RT-PCR as described in panel B except primers P25 and P31 were used for PCR amplification step. To distinguish transcripts originated from *SMN2*, PCR products were digested with DdeI [55]. *SMN1* and *SMN2* transcripts are indicted on the right of the gel. Exon 7-included (+) and exon 7-skipped (−) products are indicated on the left of the gel. The percentage of *SMN2* exon 7 skipping was calculated as in Figure 2B. ***D,*** Western blot showing translated products generated from *SMN* in GM20384 and GM03813 cells treated with PQ for 24 h. We loaded 50 and 80 µg of protein from GM20384 and GM03813 cell lysates, respectively. Primary antibodies used for probing are indicated on the left of the gel. Although the predicted molecular mass of SMN is 32 kDa, it migrates as 40 kDa band due to posttranslational modifications.

We employed MESDA to first profile major splice variants of *SMN1* and *SMN2* in GM20384 and GM03813 cells, respectively. *SMN1* in GM20384 cells produced one predominant band corresponding to the full-length transcript (Figure 3B, lane 2). In addition, *SMN1* showed very small but detectable skipping of exon 5 and exon 3. These results reveal for the first time the possibility of alternative splicing of *SMN1* exon 3 that codes for a critical tudor domain. Previous studies have shown that tudor domain plays an important role in nucleocytoplasmic trafficking of SMN [25]. GM03813 cells showed two prominent bands corresponding to the full-length and exon 7-skipped transcripts of *SMN2*. Similar to *SMN1* splice variants in GM20384 cells, we also detected skipping of *SMN2* exon 5 and exon 3 in GM03813 cells. However, unlike *SMN1*, *SMN2* showed co-skipping of exons 5 and 7 as well as exons 3 and 7 (Figure 3B, lanes 1 and 2). We also observed much weaker bands representing *Δ*3,5 and *Δ*3,5,7 transcripts in GM03813 and GM20384 cells. As controls, we used GM20383 lymphocytes and undifferentiated neuronal SH-SY5Y cells. Since these cells contain both, *SMN1* and *SMN2*, they produced a mixture of transcripts representing all *SMN* splice isoforms. Interestingly, the results of MESDA revealed almost identical splicing pattern of *SMN* in SH-SY5Y and GM20383 cells, suggesting similarity in splicing of *SMN* between neuronal and non-neuronal cells. However, compared to other cell types examined, we observed higher expression of major *SMN* splice variants in SH-SY5Y cells. Using cloning and sequencing, we next characterized the relative abundance of *SMN1* and *SMN2* transcripts for specific splice variants in SH-SY5Y cells. Some splice variants were also sequenced in other cell lines. In general, we sequenced between 8 and 17 clones from major isoforms amplified by MESDA. DdeI digestion combined with sequence analysis confirmed that isoforms lacking exon 7 came mostly from *SMN2* (Figure 4C and Figure S1). Based on sequence analysis, transcripts containing exon 7 but lacking exon 5 or exon 3 were generated mostly from *SMN1* (Figure S1). It remains to be seen if these results were affected by potentially different copy numbers of *SMN1* and *SMN2* genes in SH-SY5Y cells. Our detection of *SMN* transcript lacking exons 5 and 6 (*Δ*5,6) in GM20383 lymphocytes was a novel and an unexpected finding. This transcript is generated by an unusual pairing between the 5′ ss exon 4 and the 3′ ss exon 7. Considering that the 3′ ss *SMN1* exon 7 is much stronger than the 3′ ss *SMN2* exon 7, we expected a higher occurrence of *SMN1Δ*5,6 compared to *SMN2Δ*5,6. Indeed, sequence analysis revealed that an overwhelming majority of *Δ*5,6 transcripts generated were from *SMN1* (Figure S1A).

### Effect of OS on Splicing of *SMN* Exons

Having established that various exons of *SMN1/SMN2* are alternatively spliced, we set out to distinguish between general versus neuronal cell-specific effect of OS on *SMN* pre-mRNA splicing. To induce OS, neuronal and non-neuronal cells were treated with 1 mM PQ and transcripts were analyzed by MESDA 24 h post treatment. Of note, a previous study has shown that 1 mM of PQ is sufficient to produce a significant oxidative stress in neuronal SH-SY5Y cells [60]. We observed OS-induced increase in skipping of *SMN2* exon 7 in all cell types treated with PQ (Figure 4 and Figure S2). Based on results of DdeI digestion (Figure 4C), the strongest effect of OS on splicing of *SMN2* exon 7 was observed in GM20383 lymphocytes followed by neuronal SH-SY5Y cells. Substantial skipping of *SMN2* exon 7 was also observed in GM03813 fibroblasts treated with PQ. Based on these findings, we conclude that response to PQ-induced OS on splicing of *SMN2* exon 7 is universal. In addition, DdeI digestion analysis underscores that OS-induced skipping of exon 7 is associated mostly with *SMN2* (Figure 4C). Supporting the reduced vulnerability of *SMN1* exon 7 to skipping under OS, we detected very low levels of *SMN1* exon 7-skipped products in PQ-treated SH-SY5Y cells (Figure 4C, lane 8).

We observed an increase in PQ-induced skipping of *SMN1* exon 5 in GM20384 lymphocytes, suggesting that pre-mRNA splicing of human *SMN1* is sensitive to OS (Figure 4B). To validate that the effect of OS on *SMN1* exon 5 splicing is not specific to lymphocytes and/or related to BD-causing mutations in *CLN3* gene, we isolated and cloned RT-PCR products lacking exon 5 from PQ-treated SH-SY5Y cells. Based on sequence analysis, conditions of OS led to an appreciable increase in *SMN1Δ*5 transcripts compared to *SMN2*Δ5 (Figure S1). These results confirm that skipping of exon 5 is one of the major *SMN1*-associated events in cells undergoing OS. Unlike *SMN1*, most *SMN2* transcripts that went through exon 5 skipping also lacked exon 7. Consistently, we observed an enhanced co-skipping of *SMN2* exons 5 and 7 in PQ-treated GM03813 fibroblasts, GM20383 lymphocytes and neuronal SH-SY5Y cells (Figure 4B). Interestingly, PQ-induced OS caused a noticeable reduction in intensity of several fast migrating exon 3-skipped splice variants (Δ3, Δ3,5 and Δ3,7) in SH-SY5Y cells. Consistent with these findings, results of quantitative real-time PCR (qPCR) using various junction primers supported a decrease in skipping of exon 3 in PQ treated SH-SY5Y cells (Figure S3). However, due to a near background level of expression of Δ3 transcripts, suppression of exon 3 skipping under OS was not accompanied by an appreciable increase in the exon 3-included transcripts.

Conditions of OS produced three additional novel *SMN1* splice variants that we captured in neuronal SH-SY5Y cells (Figures 4A and 4B). The first such variant lacked *SMN1* exons 4 and 7 (*SMN1*Δ4,7) and co-migrated with the *SMN*Δ3 transcript in a polyacrylamide gel (Figure 4B). Considering skipping of constitutive exon 4 has not been previously reported and skipping of *SMN1* exon 7 is an infrequent occurrence, presence of *SMN1*Δ4,7 was very surprising. The second novel variant lacked *SMN1* exons 3 and 4 (*SMN1*Δ3,4) and co-migrated with Δ3,5,7 transcripts on a polyacrylamide gel (Figures 4A and 4B). The third novel *SMN1* splice variant that we captured lacked exons 5, 6 and 7 (*SMN1*Δ5,6,7). Occurrence of *SMN1*Δ5,6,7 reveals for the first time the feasibility of a rare phenomenon where three adjacent exons of *SMN* are skipped.

All internal exons of *SMN* are divisible by three. Thus, skipping of one or more internal exons of *SMN* does not create a premature termination codon. Consequently, none of the alternatively spliced variants of *SMN* are natural substrates of nonsense-mediated decay (NMD). To analyze the translated products of various alternatively spliced transcripts of *SMN1* and *SMN2*, we performed western blot analysis of PQ-treated GM20384 and GM03813 cells that carry *SMN1* and *SMN2*, respectively. In both cell types, we noticed a decrease in SMN levels at 24 h post PQ treatment (Figure 4D). However, we were unable to detect translated products corresponding to any of the short transcripts generated from either *SMN1* or *SMN2*. This could be attributed to several factors, including low levels of exon-skipped transcripts, slow rate of translation under the conditions of OS and unstable nature of Δ7-translated products. Of note, skipping of *SMN* exon 7 is known to create a degradation signal [21].

### Translation Efficiency of *SMN* Transcripts Lacking Internal Exons

**Figure 5:**
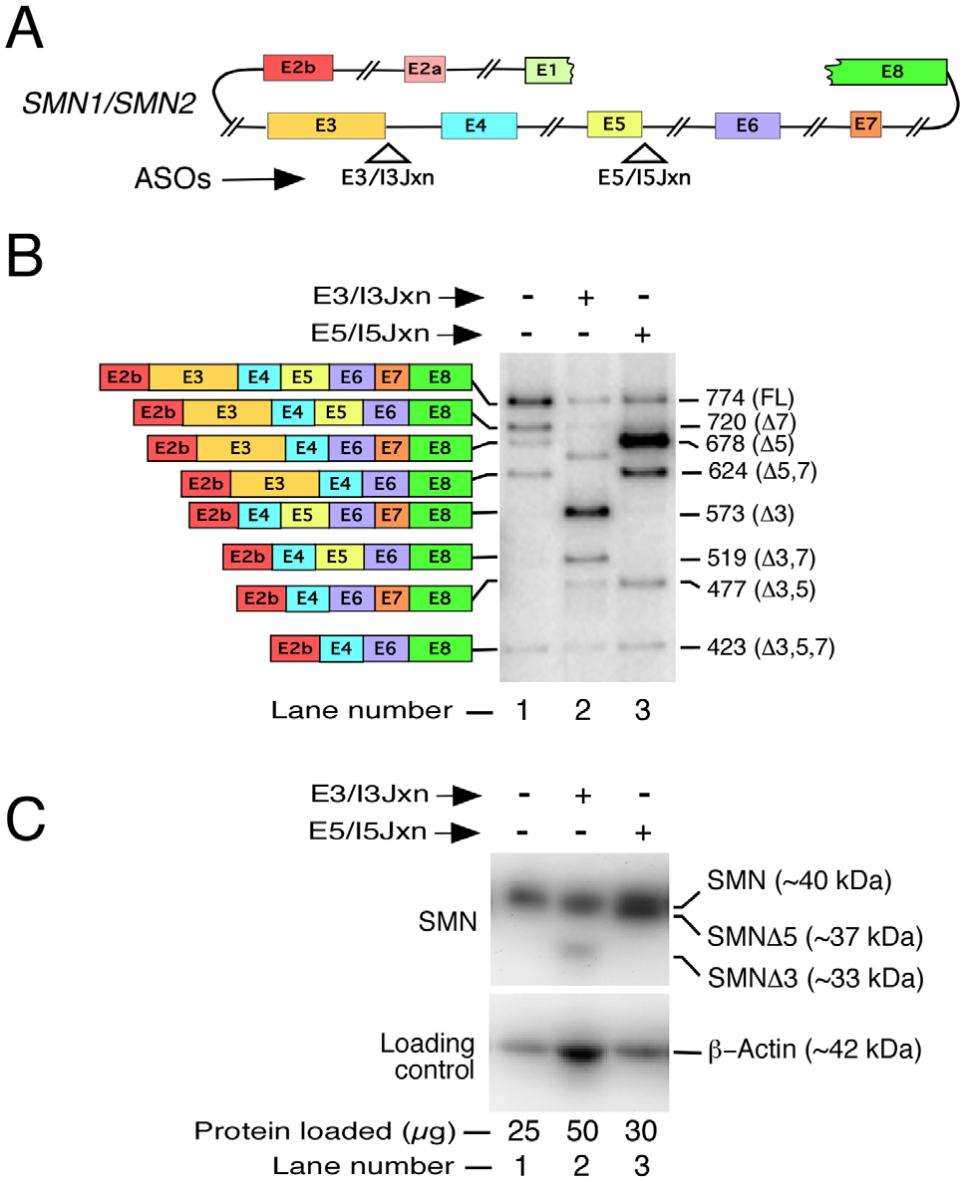
Translational efficiency of transcripts generated by ASO-induced skipping of *SMN* exons in HeLa cells. *A,* Diagrammatic representation of *SMN* pre-mRNA showing annealing positions of ASOs. Exonic and intronic sequences are depicted as in (Figure 2A). ***B,*** Splicing pattern of endogenous *SMN* in HeLa cells treated with various ASOs. Names of ASO used are given on the top of the gel. HeLa cells were transfected with 100 nM of an individual ASO. Cells were collected for total RNA preparation ∼38 hours post transfection. Spliced products were analyzed by RT-PCR as described in Figure 3B. Diagrammatic representation of spliced variants is given on the left of the gel; their sizes are indicated on the right of the gel with names of skipped exons given in brackets. All spliced products represent mixture of transcripts generated from both, *SMN1* and *SMN2*. ***C,*** Western blot showing SMN protein isoforms generated from transcripts in panel B. Cells were collected for whole-cell lysate preparation ∼38 hours post transfection. In order to capture the low abundance of SMNΔ3 isoform, we loaded 50 µg of protein from sample treated with E3/I3 Jxn ASO. Protein loading was confirmed by re-probing the blot with anti-β-actin antibodies. Primary antibodies used for probing are indicated on the left of the gel; whereas, SMN isoforms are shown on the right of the gel. SMN is present in all lanes. SMNΔ3 is detectable in lane 2, whereas SMNΔ5 is detectable in lane 3.

In order to determine whether proteins encoded by transcripts that lack one or more internal *SMN* exons could be produced, we employed an alternative approach. We induced *SMN2* exon skipping in HeLa cells using two ASOs: E3/I3Jxn and E5/I5Jxn (Figure 5A). E3/I3Jxn and E5/I5Jxn blocked the 5′ ss of exon 3 and exon 5, causing massive skipping of exon 3 and exon 5, respectively (Figure 5B). Targeting ASOs did not discriminate between *SMN1* and *SMN2* transcripts that code for identical proteins. Also, ASOs were designed not to interfere with the translational machinery because they annealed to sequences that are removed during pre-mRNA splicing. Our approach provided high levels of *SMN* exon-skipped transcripts that served as the needed templates for protein synthesis. As a result, we were able to detect proteins generated from transcripts lacking either exon 3 or 5 (Figure 5C, lanes 2 and 3). SMNΔ5 appeared to be stable and migrated very close to the full-length SMN. On the other hand, SMNΔ3 seems to be less stable. Poor stability of SMNΔ3 could be attributed to its misfolding due to the loss of the tudor-domain coded by exon 3. Our results validate that *SMN*Δ3 and *SMN*Δ5 transcripts could be translated. Whether SMNΔ3 and SMNΔ5 proteins play any physiological role is a matter of future investigation.

### Effect of Promoter on OS-induced Skipping of *SMN* Exon 7

**Figure 6:**
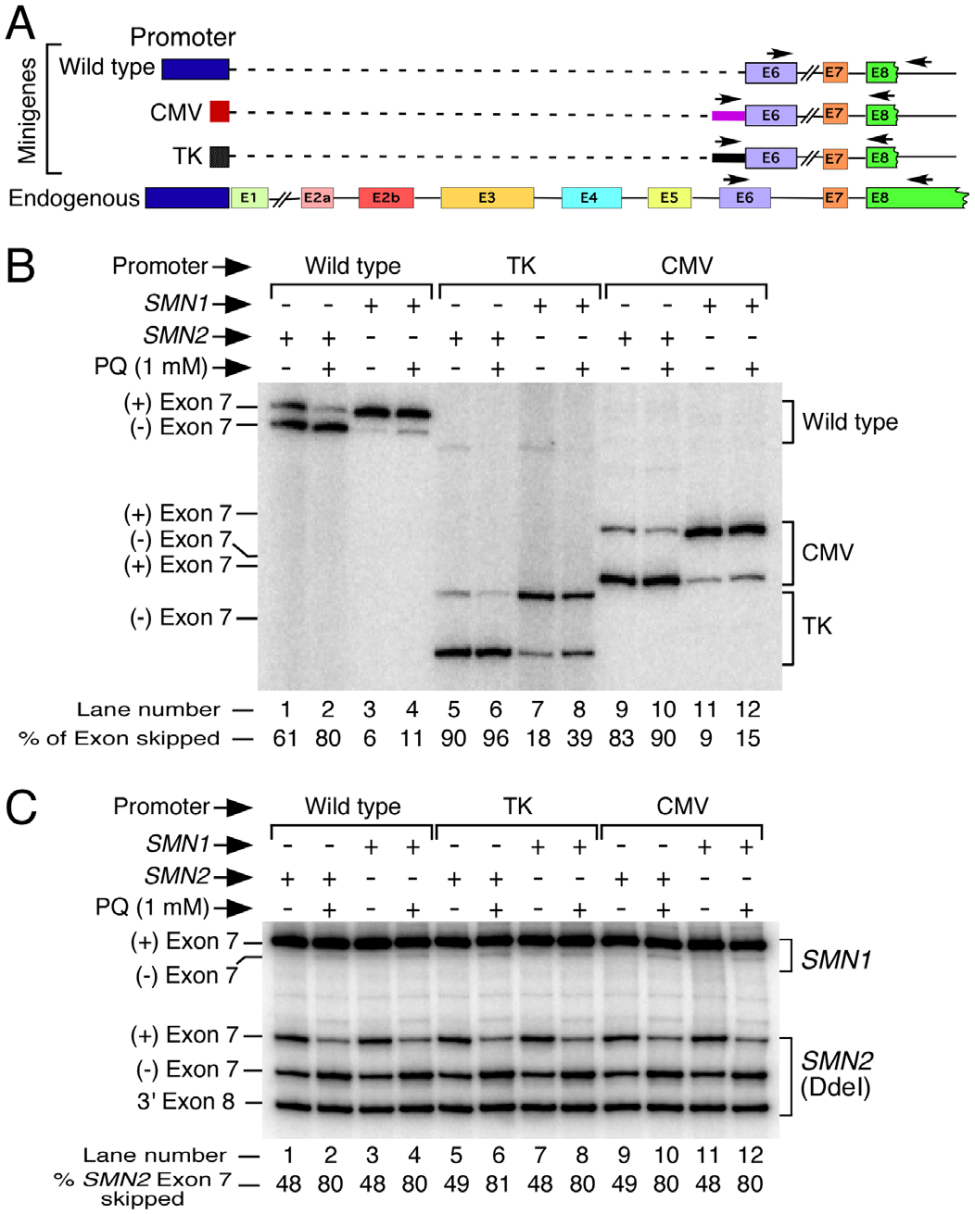
Effect of PQ on splicing of different *SMN* minigenes. *A,* Diagrammatic representation of promoter and exon/intron organization in different minigenes as compared to endogenous *SMN*. Promoters and exons are shown as boxes, introns are indicated as lines. Alternatively spliced exons 3, 5 and 7 are shown as colored boxes. Dotted lines indicate deleted sequences. Wild type refers to the minigenes under the control of human *SMN1/2* promoters; TK, minigenes under the control of thymidine kinase promoter; CMV, minigenes under the control of CMV promoter. In CMV and TK minigenes, solid bar before exon 6 refers to a vector-specific sequences (Figure S4). Locations of primer pairs for the amplification of spliced products have been marked by arrows. ***B,*** In vivo splicing pattern of different *SMN1* and *SMN2* minigenes. Data were generated using total RNA from SH-SY5Y cells transfected with 1 µg of an indicated minigene and treated with PQ for 21 h. Minigene splice products were analyzed by RT-PCR. We used 0.5 µg of total RNA for 5 µl of reverse transcription reaction and generated cDNA employing oligo(dT)_12–18_ primer. Loading was adjusted so that the intensity of bands between samples was comparable. Exon 7-included and skipped products are indicated on the left of the gel. Due to the change in location of the primer annealing sites, *SMN* spliced products generated from different vectors have variable lengths. The results were analyzed as previously described [55]. Abbreviations are same as described in panel A. ***C,*** Effect of PQ on splicing of endogenous *SMN* exon 7. Splice products were analyzed by RT-PCR as described in panel B, except we used N-24 and P26 primers for PCR to specifically amplify endogenous *SMN*. Of note, P26 anneals to exon 8 region, which is absent in minigenes [55]. To distinguish spliced variants originated from *SMN2*, PCR products were digested with DdeI [55]. The percentage of *SMN2* exon 7 skipping was calculated as described in Figure 2B. Labeling was the same as described in panel A and Figure 2B.

Promoter structure (sequence) has been shown to affect alternative splicing of specific exons in several genes including fibronectin (FN), calcitonin-gene-related product (CGRP) and CD44 [62], [63], [64]. *SMN* promoter has been localized in a 2 kb region upstream of the coding sequence that starts within exon 1 [65]. Using reporter assays, two earlier studies support similarity of promoter activity between *SMN1* and *SMN2* [65], [66]. Based on effect of small compounds that act as inhibitors of histone deacetylase 1, recent reports suggest the role of promoter sequences in splicing regulation of *SMN* exon 7 [50], [67]. However, there is no study to implicate a direct role of *SMN* promoter sequence on usage of exon 7. To address the promoter-specific splicing regulation of *SMN2* exon 7 under normal and OS conditions, we employed *SMN* minigenes with three different promoters: cytomegalovirus (CMV), thymidine kinase (TK) and wild type *SMN1*/*SMN2* promoters (Figure 6A). Several CMV promoter-containing minigenes encompassing *SMN* genomic sequences from exon 6 through exon 8 cloned in pCI vector (Promega) have been reported [19], [68], [69]. We took advantage of CMV promoter containing short minigenes that maintained an earlier reported deletion within intron 6 [68]. Due to decreased size, these minigenes provide desired benefit of high transfection efficiency without any apparent change in splicing pattern of *SMN* exon 7. To generate minigenes under the control of wild type *SMN1* and *SMN2* promoters, we replaced CMV promoter with ∼3.5 kb genomic sequences harboring promoter region of *SMN1* and *SMN2*, respectively. Wild type *SMN1* and *SMN2* promoters used in this study were the same as reported in an earlier study that confirmed similarity of transcriptional regulation between two *SMN* genes [66]. To generate TK promoter-containing minigenes, we subcloned *SMN* genomic sequences from pCI-based *SMN* minigenes into commercially available pTK-GLuc vector (New England Biolabs). Other than variations in promoter structures, vector-specific sequences downstream of promoters brought additional differences in the contexts of the three minigenes we used (Figure 6A and Figure S4). Therefore, the design of our minigene constructs allowed us to simultaneously examine the effect of promoters as well as sequences upstream of the *SMN* splicing cassette. We employed neuronal SH-SY5Y cells to examine the splicing pattern of *SMN* minigenes expressed under the control of different promoters. We simultaneously monitored the splicing pattern of exon 7 derived from endogenous *SMN1* and *SMN2*.

Among three promoters used in this study, we observed substantially higher levels of *SMN* expression with CMV promoter. For the purposes of comparison of splice variants, we adjusted loading of PCR products for different promoter samples. All minigenes expressing *SMN* under the control of various promoters recapitulated the splicing pattern of endogenous gene, with predominant exon 7 skipping in *SMN2*, and predominant exon 7 inclusion in *SMN1* (Figure 6B). However, unlike minigenes under the control of TK and CMV promoters, wild type promoter produced noticeably less exon 7 skipping (Figure 6B, lane 4). These results provide the first direct evidence of the role of promoter structure in modulation of *SMN* exon 7 splicing. However, compared to a reported 10-fold decrease in usage of the extra domain I (EDI) exon of FN when expressed under control of a CMV promoter [64], impact of *SMN* promoter on percentage of exon 7 skipping could be considered as less prominent (Figure 6B, compare lanes 1 and 2 with lanes 9 and 10). Effect of promoter structure on *SMN* exon 7 splicing was further supported by an appreciable change in the levels of exon 7 inclusion when CMV promoter was exchanged with TK promoter in *SMN* minigenes (Figure 6B, compare lanes 5 and 6 with lanes 9 and 10). Interestingly, *SMN2* transcripts derived from endogenous gene showed ∼13% less exon 7 skipping as compared to *SMN2* minigene expressed under wild type promoter (compare Figure 6B with Figure 6C). This difference could be attributed to the context of the endogenous gene, which is subjected to chromatin remodeling during transcription elongation. Considering rate of transcription elongation and transcriptional pausing affects the outcome of alternative splicing [5], [70], [71], a moderate difference in splicing of *SMN2* exon 7 between full endogenous gene and minigene is expected.

Treatment of SH-SY5Y cells with PQ caused noticeable increase in skipping of *SMN* exon 7 from all minigenes expressed under different promoters. However, distinctions between *SMN1* and *SMN2* exon 7 splicing under the conditions of OS were more pronounced in the context of endogenous promoter followed by the expression under wild type promoter. For instance, under the conditions of OS, levels of exon 7-containing *SMN2* transcripts decreased ∼2-fold and more than 2.5-fold and in the context of wild type and endogenous promoters, respectively (Figure 6B, lanes 1 and 2; Figure 6C). At the same time, levels of exon 7-containing *SMN1* transcripts in these contexts decreased only marginally. *SMN1* expressed under the control of TK promoter produced appreciable exon 7 skipping. Also, *SMN1* expressed under the control of TK promoter caused the highest levels exon 7 skipping under the conditions of OS (Figure 6B, lanes 7 and 8). Overall, our results support the role of promoter sequence in regulation of *SMN* exon 7 splicing under the conditions of OS. However, promoter sequences were not the sole regulatory elements to affect OS-induced *SMN* exon 7 splicing. Considering *SMN1* expressed under all promoters maintained the high levels (>60%) of exon 7-included transcripts even under the conditions of OS, cis-elements within *SMN* exon 7 and/or within flanking intronic sequences also contribute towards OS-induced exon 7 skipping (Figure 6B, lanes 4, 8 and 12).

### A Short ASO Targeting an Intronic GC-rich Silencer Fully Prevents OS-induced Skipping of *SMN2* Exon 7

**Figure 7:**
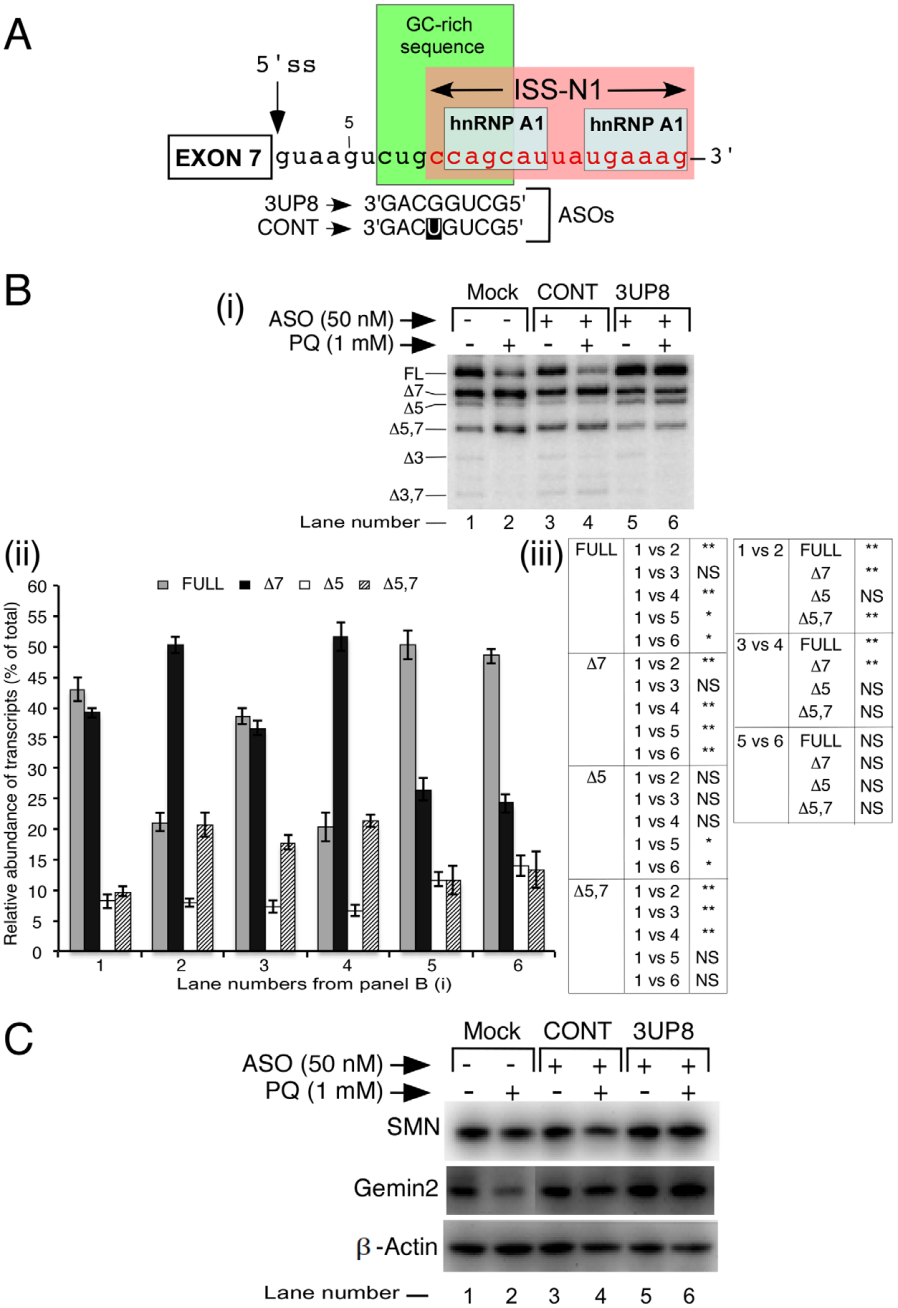
Treatment of SMA patient fibroblasts with 3UP8 ASO abrogates the negative effect of PQ. *A,* Diagrammatic representation of the ASO target area in *SMN* intron 7. Negative regulators of exon 7 splicing, GC-rich sequence, ISS-N1 and hnRNPA1 binding sites are highlighted with boxes. Annealing positions and sequences of ASOs (3UP8 and CONT) are given in 3′ to 5′ direction. 3UP8 and CONT (3UP8/64A) ASOs are the same as reported [53]. Numbering of nucleotides starts from the first position of intron 7. ***B,*** Splicing pattern of endogenous *SMN2* in SMA patient fibroblasts transfected with different ASOs in the presence (+) or absence (-) of PQ. GM03813 fibroblasts grown in 100 mm dishes were transfected with 50 nM of an ASO. About 24 hours after transfection, PQ was added to cells with a fresh growth medium at a final concentration of 1 mM. Cells were collected for total RNA/protein preparation 24 h post treatment. Spliced products were analyzed by RT-PCR. We used 1.2 µg of total RNA per 10 µl of reverse transcription reaction and generated cDNA employing 3′E8-Dde primer. Splice isoforms are indicated on the left of the gel as shown in panel (i). Mock refers to untransfected cells. Relative abundance of *SMN* spliced isoforms was calculated in each sample and is shown as a bar diagram given in panel (ii). Error bars based on standard error of the mean have been described in panel (iii). *, P<0.05; **, P<0.01; NS, not statistically significant. ***C,*** Western blot showing the levels of SMN and its interacting partner, Gemine2, in SMA patient fibroblasts transfected with 3UP8 or control (CONT) ASO in the presence (+) and absence (−) of PQ. We used 20 µg of total protein for each sample. Equal protein loading was confirmed by re-probing the blot with anti-β-actin antibodies. Primary antibodies used for probing are indicated on the left of the gel.

One of the fundamental questions in stress-related studies is to establish whether exon-specific aberrant splicing under OS is preventable. Considering the well-characterized nature of various negative cis-elements, *SMN2* exon 7 splicing offers an ideal system to test this hypothesis. We have earlier reported that a 15-nucleotide-long intronic splicing silencer N1 (ISS-N1) and an overlapping 8-nucleotide-long GC-rich sequence play critical role in *SMN2* exon 7 skipping (Figure 7A) [48], [53], [72], [73]. An 8-mer ASO (3UP8) targeting GC-rich sequence prevents *SMN2* exon 7 skipping with high target specificity without any off-target effect on splicing of other *SMN* exons [53]. Therefore, we used 3UP8 to examine whether it will alleviate the negative effect of PQ-induced OS on splicing of *SMN2* exon 7. We first treated GM03813 cells with 50 nM of 3UP8 for 24 h and then induced OS by exposing the cells to 1 mM PQ. Cells were harvested 24 h post PQ treatment and transcripts were isolated for analysis by MESDA. As shown in Figure 7B, 3UP8 was able to fully prevent *SMN2* exon 7 skipping even under PQ-induced OS. As expected, the effect of 3UP8 was exon 7 specific since this ASO did not change the splicing pattern of other *SMN2* exons. We also used a control ASO with a single mismatch mutation. The control ASO had no effect on splicing of *SMN2* (Figure 7B). To validate that the effect of 3UP8 is not due to a general stimulation of splicing machinery, we examined the splicing pattern of *Procollagen-lysine 2-oxoglutarate 5-dioxygenase 2* (*PLOD2*) exon 14 that we determined to be also affected by PQ-induced OS. 3UP8 had no stimulatory effect on splicing of *PLOD2* exon 14 (Figure S5).

Since OS affects *SMN2* exon 7 splicing the most, leading to a decrease in the production of the full-length transcripts, we next examined whether treatment with PQ has an effect on levels of SMN protein in SMA patient cells. For this, we performed western blot analysis using lysates from cells treated similarly as described in Figure 7B. Consistent with the decrease in full-length transcript, OS produced a reduction in levels of SMN (Figure 7C). However, we did not detect SMNΔ7, a truncated protein likely to be produced by translation of *SMN2* exon 7-skipped transcript, the most predominant splice variant generated under OS. This could be due to high instability of SMNΔ7 shown to contain a protein degradation signal [21]. Similar signal would affect stability of SMNΔ5,7 that could be generated by translation of the second most predominant transcript lacking exons 5 and 7. Gemin2 is a critical SMN-interacting partner responsible for the formation of SMN complex that participates in snRNP biogenesis [24]–[26]. PQ-induced decrease in SMN levels was accompanied by a similar reduction in Gemin2, suggesting a potential adverse effect of PQ on snRNP biogenesis. Remarkably, ASO treatment that restored *SMN2* exon 7 inclusion also produced increased levels of SMN and Gemin2 (Figure 7C). Considering several ASO-based strategies to correct *SMN2* splicing in SMA have been proposed [42]–[47], our findings are significant as they suggest that these strategies will retain their efficacy even under OS conditions.

## Discussion

Occurrence of aberrant splicing under the conditions of OS is an area of growing interest due to its correlation with major human diseases including cancer, cardiovascular and neurodegenerative disorders. The fundamental issue of specificity with which OS affects splicing of certain exons of particular genes in specific tissues remains poorly understood. Here, we use human spinal muscular atrophy genes (*SMN1* and *SMN2*) as a representative system to understand the impact of OS on alternative splicing of various exons of two nearly identical genes. The full-length transcripts from both genes code for SMN, an essential protein that plays a central role in gene regulation through snRNP biogenesis [24]. Skipping of any of the seven internal exons of *SMN* results in the loss of a fully functional protein that contains several overlapping domains with defined roles. Our study addresses an important question of prioritization of splicing events by which each copy of a duplicate gene responds differently to the conditions of OS.

Publically available SMA patient fibroblast cell line (GM03813) that lacks *SMN1* has been widely used for drug screening as well as for understanding transcriptional and posttranscriptional regulation of *SMN2*. However, analogous cell line to examine *SMN1*-specific transcriptional and posttranscriptional regulation has not been found. Consequently, a side-by-side comparison of the major splice variants of *SMN1* and *SMN2* has not been reported. We serendipitously discovered a BD patient cell line (GM20384) that lacked major transcripts specific to *SMN2*. Such occurrence could be due to complete or partial deletion of *SMN2* genes. The splicing pattern of *SMN1* exon 7 in GM20384 cells appeared to be identical to those observed in other cell types including BD, Parkinson’s disease, Alzheimer’s disease and neuronal SH-SY5Y cell lines, all of which carried *SMN2*. Here, we took advantage of GM20384 cell line as a model system to examine *SMN1*-specific splicing regulation.

In order to reliably capture the relative abundance of major transcripts of *SMN*, we resorted to develop MESDA. The defining feature of MESDA was the simultaneous evaluation of splicing of five internal exons (exons 3, 4, 5, 6 and 7), among which exons 3, 5 and 7 are known to be alternatively spliced. On the expected lines, GM03813 cells produced two abundantly expressed splice variants corresponding to the full-length and *SMN2* exon 7-skipped (*SMN2*Δ7) transcripts (Figure 3B, lane 1). In addition, GM03813 cells generated *SMN2*Δ*5,7* and *SMN2*Δ5 as the third and fourth most abundant transcripts, respectively. Low levels of *SMN2*Δ5 as compared to *SMN2*Δ5,7 was somewhat surprising as it suggested a cooperative mode of action in which spliced intermediates lacking *SMN2* exon 7 served as a preferred substrate for exon 5 skipping. GM03813 cells produced very low levels of *SMN2*Δ3, *SMN2*Δ3,7, *SMN2*Δ3,5 and *SMN2*Δ3,5,7 transcripts, demonstrating the feasibility of all combinations of co-skipping events of three alternate exons of *SMN2*. Among low abundant novel isoforms, we identified Δ5,6 transcript in GM20383 lymphocytes (Figure 3B, lane 3). The infrequent occurrence of this splice variant could be ascribed to a rare paring of the 5′ ss of exon 4 with the 3′ ss of exon 7, which itself is an alternatively spliced exon. Considering C6U mutation in *SMN2* creates a weak 3′ ss of exon 7, we observed substantially less *SMN2*Δ5,6 transcripts compared to *SMN1*Δ5,6 transcripts in GM20383 lymphocytes.

Beyond a handful studies reported more than a decade ago on exon 7- and exon 5-skipped transcripts [18], [19], [58], our understanding of transcript diversity generated by endogenous *SMN1* remains very limited. Therefore, several of our findings reported here on *SMN1* splicing constitute a significant advancement towards a better understanding of an overall posttranscriptional regulation of *SMN1*, which serves as the primary source for maintaining healthy SMN levels in general population. Our results established that the skipping of *SMN1* exons 3 and 5 is a general phenomenon that occurs in neuronal and non-neuronal cells alike. Based on the conserved nature of a sequence spanning from exon 2a through exon 6 of *SMN* genes, one could speculate that the mechanism of splicing of exons 3 and 5 is the same for *SMN1* and *SMN2*. However, lack of *SMN1*Δ5,7 transcripts owing to the absence of *SMN1* exon 7 skipping was not accompanied by a proportionate gain in *SMN1*Δ5 transcripts, suggesting that inclusion of *SMN1* exon 7 has a favorable effect on inclusion of exon 5. This could be due to the supporting role of a new sequence and/or structural context created by the inclusion of exon 7. Interestingly, we observed about 13-fold more *SMN1* exon 3 skipping as compared to *SMN2* exon 3 skipping in neuronal SH-SY5Y cells. Also, as compared to *SMN2*Δ3 transcripts, less proportion of *SMN1*Δ3 transcripts underwent through co-skipping with exon 5 (Figure S1). These results suggest an inverse correlation between skipping events of exon 3 and exon 5 of *SMN1*. Our subsequent finding that PQ-induced enhanced skipping of *SMN1* exon 5 suppresses generation of Δ3 transcripts supports such mechanism (Figure 4B). It remains to be seen if such correlation is due to the predominant inclusion of exon 7 in *SMN1*.

Effect of PQ-induced OS on splicing in different cell types (GM03813, GM20384, GM20383 and SH-SY5Y) revealed remarkable similarities as well as differences between two *SMN* genes. Supporting an earlier report [60], PQ-induced OS produced a significant skipping of *SMN2* exon 7, whereas *SMN1* exon 7 splicing remained mostly unaffected (Figure 4C). However, the sensitivity of our assay combined with the cell types used demonstrated that high susceptibility of *SMN2* exon 7 to skipping under the conditions of OS is more general than previously thought. Consistently, all *SMN2*-containing cells in our study showed substantial *SMN2* exon 7 skipping under conditions of OS. We show that OS-induced skipping of *SMN2* exon 5 happens primarily (if not exclusively) as co-skipping of *SMN2* exons 5 and 7 (Figure 4B, lane 2). By contrast, skipping of *SMN1* exons 5 under conditions of OS takes place almost always without skipping of *SMN1* exon 7 (Figure 4B, lane 4). Our results also demonstrate that OS-induced skipping of *SMN* exon 5 occurs in both, neuronal and non-neuronal cells. Exon 5 of *SMN* codes for a recently described proline-rich calpain cleavage domain [27]. Owing to the low levels of *SMN1*Δ5 transcripts, we could not detect SMNΔ5 protein under the conditions of OS (Figure 4D). However, our finding that SMNΔ5 is stably translated is significant (Figure 5C). Future studies will address if the calpain cleavage domain lacking protein (SMNΔ5) generated by *SMN1*Δ5 has any physiological significance.

Skipping of any of the internal exons of *SMN* maintains the reading frame. Therefore, NMD pathway that degrades mRNAs carrying a premature termination codon is not applicable for the reduced levels of any of the short *SMN* transcripts. We detected three novel *SMN1* isoforms (*SMN1*Δ4,7; *SMN1*Δ3,4 and *SMN1*Δ5,6,7) generated under the conditions of OS. It is not known if low abundance of these splice variants are in part due to their poor stability caused by a non-NMD mechanism. Presence of *SMN1*Δ4,7 underscores the occurrence of a rare splicing event of exon 4 skipping in which the 5′ ss of exon 3 and the 3′ ss of exon 5 are required to pair. Considering exons 3 and 5 are also alternatively spliced, skipping of exon 4 represents a unique event that guarantees promotion of inclusion of both, exons 3 and 5. Therefore, our finding of *SMN1*Δ4,7 reveals the first mutually exclusive event with a significance to the prevention of skipping of two alternatively spliced exons of *SMN1*. Also, generation of *SMN1*Δ4,7 comes at the expense of competing events that lead to production of two novel isoforms: *SMN1*Δ3,4 and *SMN1*Δ5,6,7. Presence of *SMN1*Δ5,6,7 underscores a unique splicing event requiring a rare long-distance pairing between the 5′ ss of exon 4 and the 3′ ss of exon 8. Another significant observation of our study was the stimulatory effect of OS on splicing of exon 3 (Figures S2 and S3). It remains to be seen if decrease in exon 3 skipping under the conditions of OS contributes at least in part towards generation of some of the novel splice variants describe above.

Increasing evidence support transcription-coupled splicing regulation. Effect of transcription on alternative splicing could be exerted through transcription initiation at specific promoters as well as through transcriptional pausing [5]. Well-known factors that affect alternative splicing in a promoter-specific manner include steroid hormone nuclear receptor coactivators, human papilloma virus (HPV) transcriptional activator E2 and peroxisome proliferator-activated receptor coactivator-1α (PGC-1α) [64]. Our finding that large wild type promoter sequence in our reporter system suppresses skipping of *SMN* exon 7 provided the first direct evidence of the role of promoter in regulation of *SMN* exon 7 splicing. Effect of promoter sequence on regulation of *SMN* exon 7 splicing was also observed under the conditions of OS. In particular, OS-induced differential splicing regulation between *SMN1* and *SMN2* was much more apparent in the context of the wild type promoter as compared to CMV and TK promoters. Decreased ATP level in OS is likely to slow down or even pause transcription elongation with a significant consequence to ss selection and exon usage [70], [71]. Generation of a long endogenous transcript requires an extensive transcription elongation step. Therefore, it is likely that the effect of OS is exerted mostly at the level of transcription elongation. Our finding that endogenous *SMN2* produced the highest degree of exon 7 skipping under the conditions of OS also supports the role of transcription elongation in OS-induced splicing regulation of *SMN2* exon 7. Recently, transcriptional elongation regulator 1 (TCERG1) has been found to regulate alternative splicing of the short isoform of B-cell lymphoma-extra (BCL-x_s_) [74]. It remains to be seen if analogous mechanism accounts for the regulation of *SMN2* exon 7 splicing under the conditions of OS. However, our results do not preclude the role of additional factors that act through transcription initiation albeit variably at different promoters expressing *SMN* minigene under the conditions of OS.

Translation of specific transcripts is selectively affected under the conditions of stress [75]. Our finding that ASO-mediated prevention of *SMN2* exon 7 skipping under the conditions of OS is able to restore the levels of SMN and Gemin2 suggests that OS does not affect selective repression of *SMN* translation. Given the prominent role of SMN in cellular metabolism, it is imperative that cells maintain a minimum SMN level even under the conditions of OS. Selective skipping of exon 7 from one gene (*SMN2*) but not from the other (*SMN1*) supports this argument. Although Δ7 and Δ5,7 were the most abundant *SMN2* transcripts generated under OS, we could not detect their corresponding translated products. This could be due to a protein degradation signal coded by the exon 7-skipped transcripts [21]. Our ASO-based approach ruled out an analogous degradation mechanisms for the translated product generated from Δ3 and Δ5 splice variants. Cells prioritize mRNA translation and storage under stress-associated conditions [75]. Hence, it is possible that shorter splice variants of *SMN* served as decoy molecules to capture microRNAs (miRNAs) and relieve full-length transcripts of miRNA repression. This mechanism will allow a better synthesis of SMN even from the low levels of full-length transcripts. A recent report provides a strong proof of principle for such mechanism in an analogous system [76]. miRNA-associated translational repression is generally associated with the 3′ untranslated regions (UTRs) [77]. However, it (repression) could also occur through targets within isoform-specific coding sequences [77], [78]. Given the fact that PQ treatment generates an altered 3′ UTRs due to overwhelming skipping of the last coding exon (exon 7) and also produces additional splice variants, there is a plausible possibility of miRNA-associated control of SMN levels in stress-associated conditions. Now that we have confirmed the vulnerability of various *SMN* exons to skipping under the conditions of OS, future experiments would address the mechanism of OS-induced aberrant splicing regulation of *SMN* and the physiological role of various *SMN* transcripts generated under stress-associated conditions.

In summary, our findings uncover the surprising diversity of *SMN* transcripts expressed under normal and OS conditions. We validate our findings employing several complementary approaches including MESDA and a unique cell type devoid of major *SMN2* transcripts. Our findings underscore an added vulnerability of SMA patients to the conditions of OS and demonstrate the efficacy of an ASO-based strategy in splicing correction under OS. Our results provide the first direct evidence of role of *SMN* promoter sequence in regulation of *SMN* exon 7 splicing under normal and OS conditions. In addition to a better understanding of SMA pathogenesis, our findings bring new perspective to splicing regulation of a model housekeeping gene associated with one of the leading genetic causes of infant mortality.

## Materials and Methods

### Plasmid Constructs

Minigene splicing cassettes pSMN1ΔI6 and pSMN2ΔI6 have been described previously [68]. Designs of newly constructed minigenes and primer sequences for cloning are given in supporting data (Table S1, Figure S4). Briefly, pTK-SMN1ΔI6 and pTK-SMN2ΔI6 constructs, in which *SMN* minigene sequences were placed under the control of Herpes Simplex Virus (HSV) thymidine kinase (TK) promoter, were generated as follows. *SMN1* and *SMN2* sequences were amplified by polymerase chain reaction (PCR) using pSMN1ΔI6 and pSMN2ΔI6 as templates, respectively. The amplification was performed with Phusion DNA Polymerase (New England Biolabs) and a pair of primers, P42 and P43. PCR products were then digested with HindIII and BamHI and inserted into pTK-GLuc vector (New England Biolabs) treated with the same restriction enzymes. To generate splicing cassettes pWTP-SMN1 and pWTP-SMN2 in which *SMN1* and *SMN2* minigene sequences were placed under the control of human *SMN1* and *SMN2* promoters, respectively, we started with PCR amplification of the *SMN1* and *SMN2* promoter sequences (∼3.5 kb) using T1 and C1 clones, respectively [66]. The promoter sequences were amplified using Phusion DNA Polymerase and primers 5′SMN-pro-BglII and 3′SMN-pro-XhoI. The amplified *SMN1* and *SMN2* promoter sequences were subsequently digested with BglII and XhoI and ligated into pSMN1ΔI6 and pSMN2ΔI6 minigenes subjected to partial digestion with the same restriction enzymes. Partial digestion was used since pSMN1ΔI6 and pSMN2ΔI6 minigenes contain two BglII restriction site. For partial digestion several µg of pSMN1ΔI6/pSMN2ΔI6 were treated with XhoI (6.4 U) at 37°C overnight; next morning BglII (4 U) was added to the reaction mixture and digestion continued for another 15 min followed by phenol:chloroform extraction and ethanol precipitation. The precipitated products were separated on a 1% agarose gel, the band of interest corresponding to 4.2 kb was excised from the gel and the DNA product was recovered from the gel using QIAquick Gel Extraction Kit (Qiagen) as per manufacturer’s instructions. The recovered DNA corresponded to either BglII- and XhoI-digested pSMN1ΔI6 or pSMN2ΔI6, which, as mentioned above, were used in ligation reaction with *SMN1* and *SMN2* promoters and Quick Ligation Kit (New England Biolabs). The identity of novel minigene clones was verified by sequencing. All primers were obtained from Integrated DNA Technologies (Table S1). All restriction enzymes used were from New England Biolabs.

### Cell Culture and ASOs

All tissue culture media and supplies were purchased from Life Technologies. Human neuroblastoma SH-SY5Y cells were cultured in 1∶1 mixture of Minimum Essential Medium (MEM, catalog # 11095) and F12 medium supplemented with 10% fetal bovine serum (FBS). Primary patient fibroblasts, immortalized B-lymphocytes as well as cells from healthy individuals were obtained from Coriell Cell Repositories (CCR). Description of cells from CCR used in this study is given in Table 1. These cells were grown according to the provided instructions. In brief, all primary fibroblasts were grown in minimal essential medium (MEM, catalog # 10370) supplemented with 2 mM GlutaMAX-I and 15% FBS. Only in case of primary fibroblasts from Alzheimer patient (Repository number AG04159), the amount of FBS in growth medium was 10%. B-lymphocytes were grown in RPMI1640 (catalog # 11875) medium supplemented with 15% FBS. The following ASOs were used in this study: 3UP8, 5′-mG*mC*mU*mG*mG*mC*mA*mG-3′; 3UP8/64A (CONT), 5′-mG*mC*mU*mG*mU*mC*mA*mG-3′; E3/I3Jxn, 5′-mU*mA*mU*mC*mC*mU*mU*mA*mC*mC*mU*mC*mU*mU*mG*mA*mG*mC*mA*mU-3′; E5/I5Jxn, 5′-U*mU*mU*mA*mC*mU*mU*mA*mC*mU*mG*mG*mU*mG*mG*mU*mC*mC*mA*mG-3′. All ASOs were obtained from Dharmacon Inc. They incorporated 2′-O-methyl modification (indicated by “m”) and phosphorothioate backbone (indicated by *) as described earlier [48].

### PQ Treatment

PQ (Paraquat, methyl viologen dichloride hydrate, catalog # 856177) was obtained from Sigma. PQ treatment of pre-plated adherent cells was done as follows. Sixteen to eighteen hours before the treatment GM03813 cells were plated at a density of ∼1.1×10^5^ cells per well of 6-well plates or ∼6.2×10^5^ per 100 mm tissue culture dish. For SH-SY5Y, ∼1.9×10^6^ and 0.5×10^6^ SH-SY5Y cells were seeded in 100 mm dishes. In case of SH-SY5Y, cells plated at a higher density were subjected to PQ treatment, while cells seeded at a lower density served as an untreated control. Lower cell density insured that untreated control SH-SY5Y cells do not become confluent at the end of PQ treatment. PQ was added with fresh growth medium at a final concentration of 1 mM; fresh medium without PQ was also added to control untreated cells. 200 mM stock solution of PQ in phosphate buffered saline (PBS) was prepared immediately before usage. In case of B-lymphocytes that grow in suspension as aggregates, right before PQ addition cell clumps were disaggregated by pipetting and cells were seeded at a density of ∼1.4×10^5^ cells per ml in a desired volume. PQ was added to a final concentration of 1 mM. In case of untreated control lymphocytes, PQ was omitted from growth medium. Unless noted otherwise, PQ treatment continued for 24 h, after which cells were collected for whole cell lysates/total RNA preparation.

PQ treatment combined with transfection of minigenes was done as follows. SH-SY5Y cells were plated at a density of ∼2.8×10^5^ cells per one well of a 6-well plate. Next day the cells were transfected with 1 µg of different minigenes using Lipofectamine 2000 (Life Technologies) following the manufacturer’s recommendations. Six hours after transfection cells were washed once and conditioned medium harvested from growing SH-SY5Y cells was added to the wells. Twelve hours post transfection, cells in each well were trypsinized and collected in a total volume of 4 ml; 2.5 ml of this cell suspension were transferred to a new well of 6-well plate and freshly prepared PQ was immediately added to a final concentration of 1 mM (PQ treated cell), while 1.5 ml of this suspension were plated in another well of 6-well plate and used as an untreated control. Following 21 hours of PQ treatment, cells were collected for total RNA preparation.

PQ treatment combined with transfection of ASOs was done as follows. GM03813 primary fibroblasts were plated at a density of ∼8×10^5^ cells per 100 mm tissue culture dish. Total number of six dishes was seeded. Twenty-four hours later cells were transfected with 50 nM of a given ASO (two plates were transfected with each ASO) using Lipofectamine 2000 following the manufacturer’s recommendations. Following another 24 hours the medium was changed, and freshly prepared PQ was added to one of each pair of transfected plates; the other plate served as a PQ untreated control. PQ treatment continued for 24 hours, after what cell were washed several times with ice-cold PBS and collected by scrapping. For total RNA preparation ∼1/4 of cells from each dish was used, while 3/4 were used for making cell lysates.

### ASO Transfections

Delivery of ASOs into HeLa cells was done by reverse transfection using Lipofectamine 2000. Complexes of Lipofectamine 2000 and ASOs were prepared following the manufacturer’s recommendations. The complexes were allowed to form for 20 minutes at room temperature, after what each complex (1 ml, in Opti-MEM I Reduced Serum Medium) was mixed with 4 ml of HeLa cell suspension in a 60 mm dish. Total number of HeLa cells used for transfection varied from ∼2.0×10^6^ to ∼2.5×10^6^ cells. The final ASO concentration was 100 nM. Next day culture medium was changed for the fresh one. About 38 hour post transfection the cells were washed several times with ice-cold PBS and collected by scraping. About 4/5^th^ of cells were used for making cell lysates, while 1/5^th^ of cells were used for preparing total RNA.

### RT-PCR

For reverse transcription and PCR (RT-PCR), total RNA was isolated using Trizol reagent (Life Technologies) following the manufacturer’s recommendations. To generate cDNA, reverse transcription was carried out using a SuperScript III reaction kit (Life Technologies). For cDNA synthesis random primers (Promega), an oligo(dT)_12–18_ primer (Life Technologies) or gene-specific primer 3′E8-Dde were employed. Generally, 0.5 to 1 µg of total RNA was used per 5 µl of Reverse transcriptase (RTase) reaction. Minigene-specific spliced products were amplified using Taq DNA polymerase and the following primer combinations: P1 and P2 for SMNΔI6 minigenes [68], P43 and P45 for pTK vector-based minigenes, and P31 and 3′Jun E8/bb for minigenes with SMN promoters. For PCR amplification of endogenous *SMN* the following pairs of primers were used: either N-24 and P26 or P25 and P31 for exon 7 splicing [55]; 5′hSMN-E2b and P2–2 for MESDA. Of note, there were no differences in endogenous *SMN* splicing pattern whether cDNA produced with oligo(dT)_12–18_ or with a gene specific primer was used for MESDA. PCR reactions were performed either in the presence of a trace amount of [α-^32^P] dATP (3,000 Ci/mmole; Perkin-Elmer Life Sciences) or with the 5′-end-P^32^-labeled primer, 5′hSMN-E2b. End-labeling of 5′hSMN-E2b was done using γ-ATP (3,000 Ci/mmole, Perkin-Elmer Life Sciences) and T4 Polynucleotide Kinase (New England Biolabs). To distinguish splice isoforms originated from *SMN2*, PCR products amplified with N-24 and P26 or P25 and P31 were subjected to overnight DdeI digestion, followed by phenol:chloroform extraction and ethanol precipitation [55]. In all cases PCR products were resolved on a 6% native polyacrylamide gel. Analysis and quantifications of splice products were performed using a FPL-5000 Image Reader and Multi Gauge software (Fuji Photo Film Inc). Results were confirmed by at least three independent experiments. All primer sequences used in RT-PCR are given in supporting data (Table S1).

### Western Blot Analysis

Whole-cell extracts from HeLa, GM20384 and GM03813 were prepared using ice-cold RIPA buffer (Boston BioProducts) supplemented with either Complete EDTA-free protease inhibitor cocktail (Roche Applied Science) or Halt Protease Inhibitor Single-Use cocktail (Thermo Scientific). Protein concentrations were determined using BCA protein assay kit (Thermo Scientific). Protein samples were resolved on an SDS-polyacrylamide gel (11 or 12%) and transferred on polyvinylidene fluoride membrane (Bio Trace PVDF, Pall Life Sciences). The following primary and secondary antibodies were used: mouse monoclonal anti-SMN (BD Transduction Laboratories), mouse monoclonal anti-Gemin2 (Sigma-Aldrich), rabbit polyclonal anti-actin (Sigma-Aldrich), horseradish-peroxidase-conjugated secondary antibodies against mouse (Jackson ImmunoResearch) and rabbit (GE Healthcare). After probing for SMN, membranes were stripped (15 min at room temperature) using Restore Western Blot Stripping Buffer (Thermo Scientific) and re-probed for β-actin. Immunoreactive proteins were visualized with SuperSignal West Dura Extended Duration Substrate or SuperSignal West Femto Maximum Sensitivity Substrate (Thermo Scientific). The membranes were scanned using UVP BioSpectrum AC Imaging System (UVP). Results were confirmed by at least three independent experiments.

### Statistical Analysis

All calculations were performed in Excel (Microsoft Office 2011 edition). Data were expressed as mean±standard error of the mean (SEM). Statistical analyses were performed using the unpaired Student’s t-test. Unless otherwise mentioned P values were two-tailed and the level of statistical significance was set at P<0.05.

## Supporting Information

Figure S1 **Distribution of splice forms revealed by cloning and sequencing.** ***A*** **,** Relative abundance of *SMN* splice isoforms. All splice variants except Δ5,6 were cloned from SH-SY5Y cells. Splice variant Δ5,6 was cloned from GM20383 cells. ***B*** **,** Evaluation of *SMN* splice isoforms generated under OS caused by PQ treatment of SH-SY5Y cells. Cells were treated with 1 mM PQ for 24 hours and analyzed by RT-PCR, cloning and sequencing.
(DOCX)

Figure S2 **Pictogram showing relative abundance of transcripts generated in various cell types undergoing through OS.** Peak values are derived from the band intensities in lanes shown in Figure 4B. Pictograms were generated by MultiGauge software version 3.0 (FUJIFILM). Abbreviation: FL, Full-length transcript.
(DOCX)

Figure S3 **Comparison of different splice variants in PQ treated and untreated SH-SY5Y cells as determined by qRT-PCR.** ***A,*** Relative expression of splice isoforms from control untreated and 1 mM PQ treated cells. qRT-PCR was performed as described in Materials and Methods S1. Total *SMN* from untreated SH-SY5Y was used for normalization. The values are mean ± SD. Abbreviations for transcripts: Total, total *SMN*; 7+, exon 7-included; Δ7, exon 7 skipped; 5+, exon 5-included; Δ5, exon 5 skipped; 3+, exon 3-included; Δ3, exon 3 skipped. *, P<0.05; **, P<0.01. ***B,*** Log2 transformed fold changes in *SMN* splice isoform levels between control untreated and 1 mM PQ treated calculated based on the results shown in Panel (A). The values are mean ± SD. Abbreviations for transcripts: Total, total *SMN*; 7+, exon 7-included; Δ7, exon 7 skipped; 5+, exon 5-included; Δ5, exon 5 skipped; 3+, exon 3-included; Δ3, exon 3 skipped. *, P<0.05; **, P<0.01.
(DOCX)

Figure S4 **Diagrammatic representation of minigenes placed under the control of different promoters.** ***A,*** *SMN1* and *SMN2* minigenes (pSMN1ΔI6 and pSMN2ΔI6) under the control of CMV promoter. Cloning strategy is described earlier [61]. ***B,*** *SMN1* and *SMN2* minigenes (pTK-SMN1ΔI6 and pTK-SMN2ΔI6) under the control of TK promoter were generated by subcloning into pTK-GLuc vector (New England Biolabs). Cloning strategy is described in Materials and methods. ***C,*** *SMN1* and *SMN2* minigenes (pWTP-SMN1ΔI6 and pWTP-SMN2ΔI6) under the control of wild type *SMN* promoters were generated by replacing CMV promoter with human *SMN1* and *SMN2* promoters, respectively. Cloning strategy is described in Materials and Methods.
(DOCX)

Figure S5 **Results showing specificity of 8-mer ASO targeting GC-rich sequence.** ***A,*** Diagrammatic representation of the ASO target area as described in Figure 7A. ***B,*** Splicing pattern of endogenous *PLOD2* in SMA patient fibroblasts transfected with different ASOs in the presence (+) or absence (−) of PQ. Transfection, PQ treatment and RNA isolation procedures were the same as described in Figure 7. *PLOD2* spliced products were analyzed by RT-PCR. RNA was converted to cDNA using an oligo(dT)_12–18_ primer. *PLOD2* transcripts were amplified using E13-PLOD2 and E15-PLOD2 primers that anneal to exons 13 and 15, respectively. Primer sequences are given in Table S1.
(DOCX)

Table S1 **List of primers used in this study.**
(DOCX)

Materials and Methods S1 **Detailed information on methods used in this study.**
(DOCX)
