# Supplementary figures and images for: A Multi-Exon-Skipping Detection Assay Reveals Surprising Diversity of Splice Isoforms of Spinal Muscular Atrophy Genes
Source: PLoS One. 2012 Nov 19;7(11):e49595. doi: 10.1371/journal.pone.0049595 (PMC3501452; doi:10.1371/journal.pone.0049595)

**Figure S1**


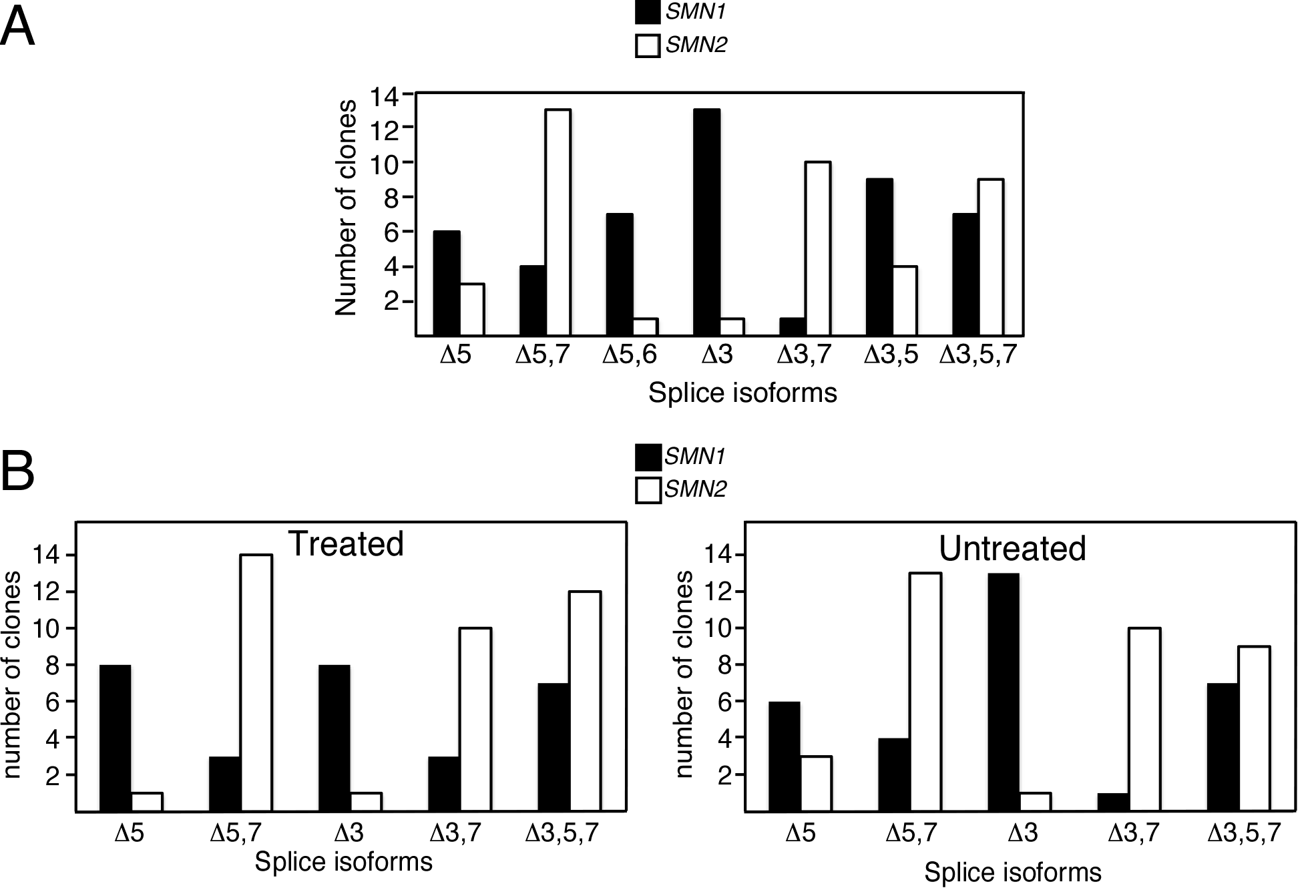

Supplement: Figure S1 — Distribution of splice forms revealed by cloning and sequencing. A , Relative abundance of SMN splice isoforms. All splice variants except Δ5,6 were cloned from SH-SY5Y cells. Splice variant Δ5,6 was cloned from GM20383 cells. B , Evaluation of SMN splice isoforms generated under OS caused by PQ treatment of SH-SY5Y cells. Cells were treated with 1 mM PQ for 24 hours and analyzed by RT-PCR, cloning and sequencing. (DOCX) [file pone.0049595.s001.docx]

**Figure S2**

**
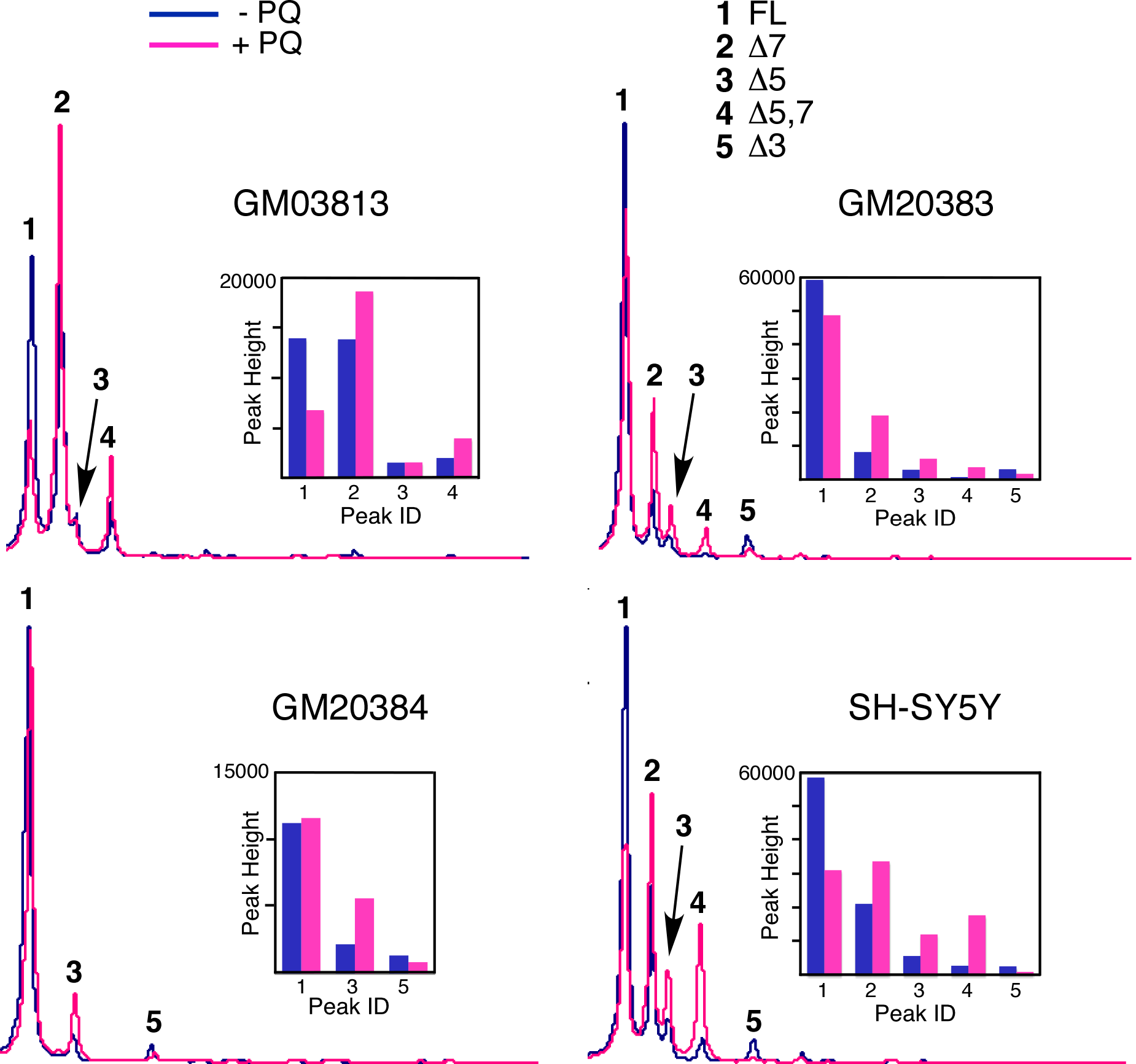
**

Supplement: Figure S2 — Pictogram showing relative abundance of transcripts generated in various cell types undergoing through OS. Peak values are derived from the band intensities in lanes shown in Figure 4B. Pictograms were generated by MultiGauge software version 3.0 (FUJIFILM). Abbreviation: FL, Full-length transcript. (DOCX) [file pone.0049595.s002.docx]

**Figure S3**


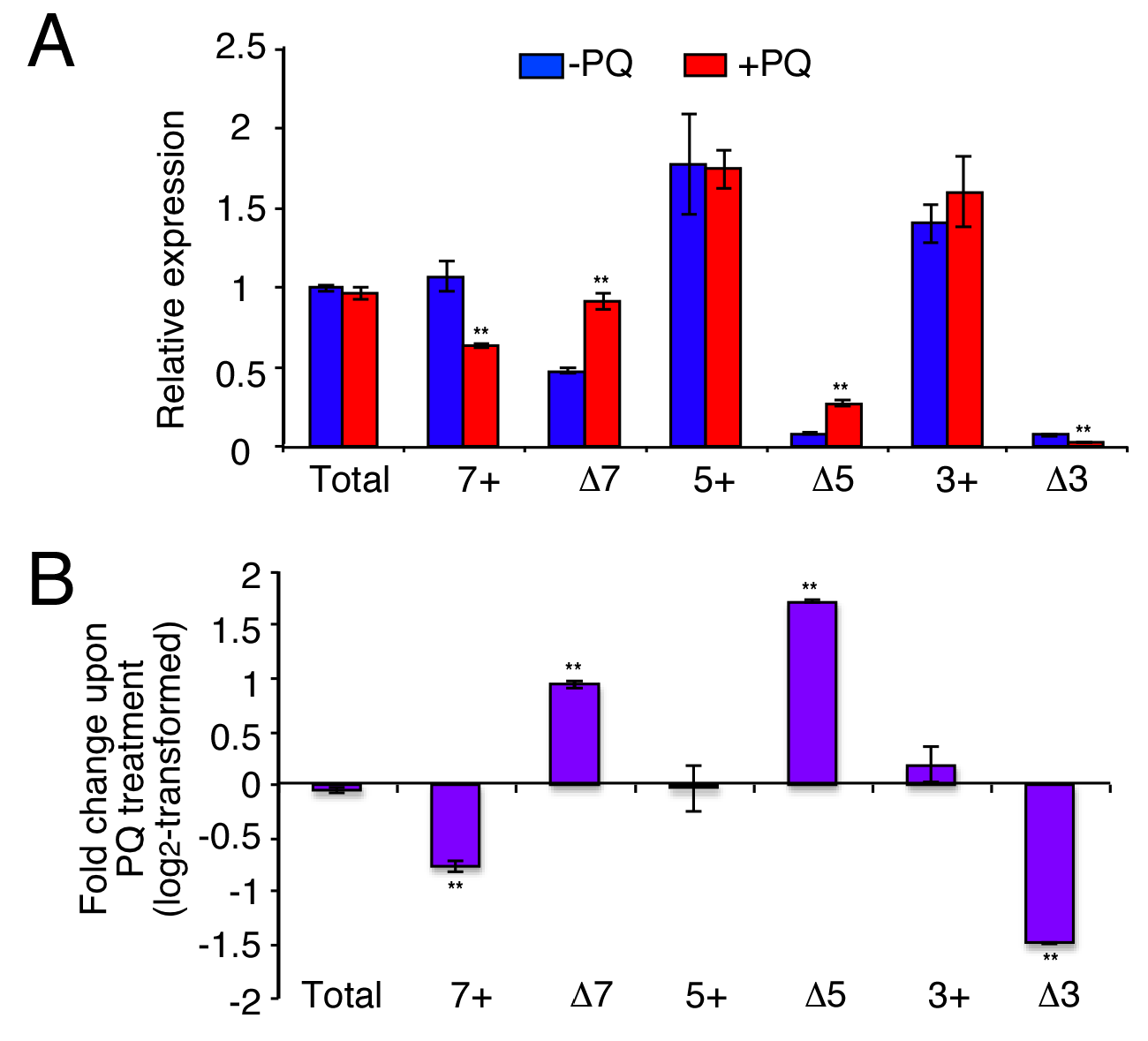

Supplement: Figure S3 — Comparison of different splice variants in PQ treated and untreated SH-SY5Y cells as determined by qRT-PCR. A, Relative expression of splice isoforms from control untreated and 1 mM PQ treated cells. qRT-PCR was performed as described in Materials and Methods S1. Total SMN from untreated SH-SY5Y was used for normalization. The values are mean ± SD. Abbreviations for transcripts: Total, total SMN; 7+, exon 7-included; Δ7, exon 7 skipped; 5+, exon 5-included; Δ5, exon 5 skipped; 3+, exon 3-included; Δ3, exon 3 skipped. *, P<0.05; **, P<0.01. B, Log2 transformed fold changes in SMN splice isoform levels between control untreated and 1 mM PQ treated calculated based on the results shown in Panel (A). The values are mean ± SD. Abbreviations for transcripts: Total, total SMN; 7+, exon 7-included; Δ7, exon 7 skipped; 5+, exon 5-included; Δ5, exon 5 skipped; 3+, exon 3-included; Δ3, exon 3 skipped. *, P<0.05; **, P<0.01. (DOCX) [file pone.0049595.s003.docx]

**Figure S4**


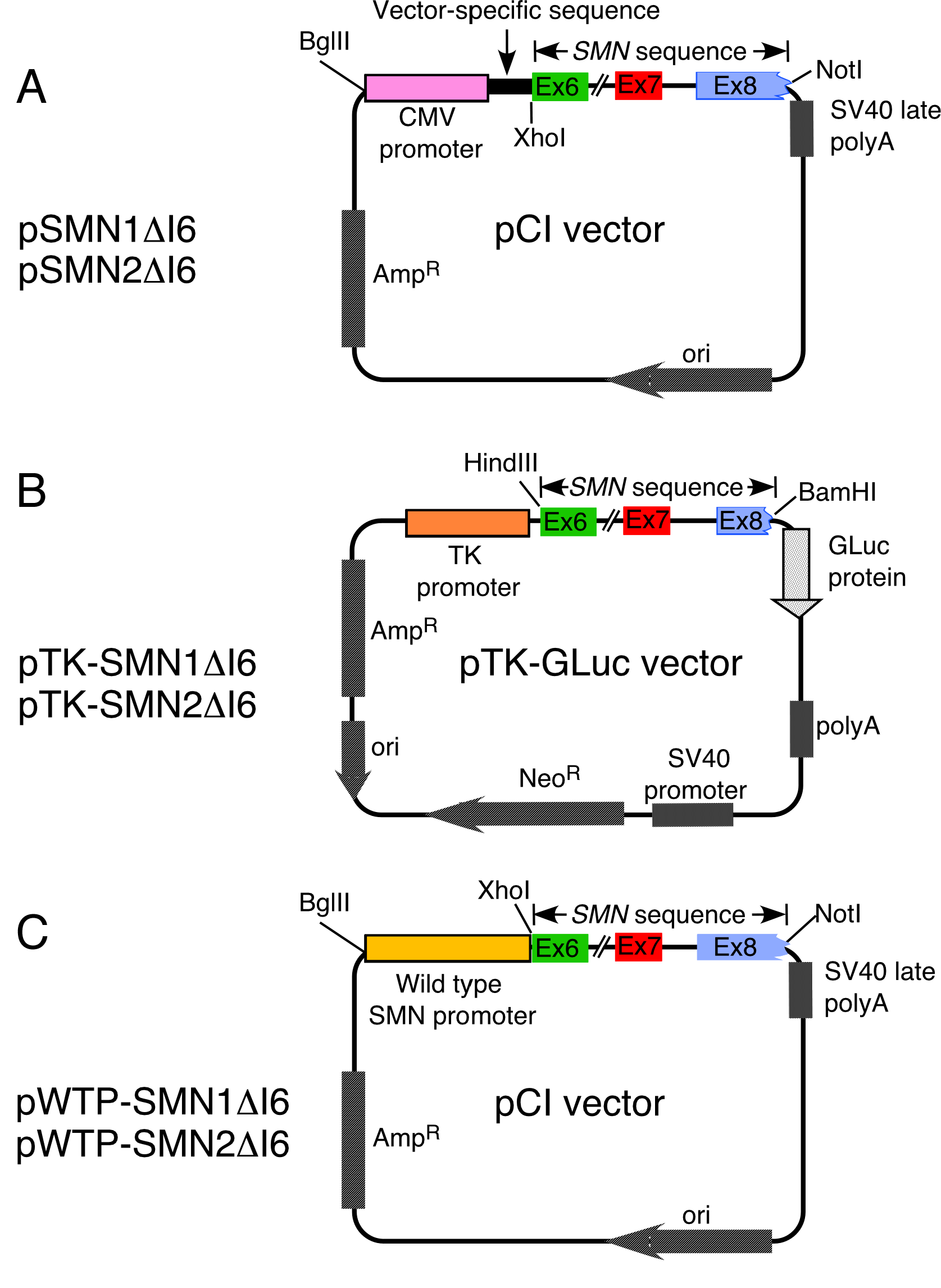

Supplement: Figure S4 — Diagrammatic representation of minigenes placed under the control of different promoters. A, SMN1 and SMN2 minigenes (pSMN1ΔI6 and pSMN2ΔI6) under the control of CMV promoter. Cloning strategy is described earlier [61]. B, SMN1 and SMN2 minigenes (pTK-SMN1ΔI6 and pTK-SMN2ΔI6) under the control of TK promoter were generated by subcloning into pTK-GLuc vector (New England Biolabs). Cloning strategy is described in Materials and methods. C, SMN1 and SMN2 minigenes (pWTP-SMN1ΔI6 and pWTP-SMN2ΔI6) under the control of wild type SMN promoters were generated by replacing CMV promoter with human SMN1 and SMN2 promoters, respectively. Cloning strategy is described in Materials and Methods. (DOCX) [file pone.0049595.s004.docx]

**Figure S5**


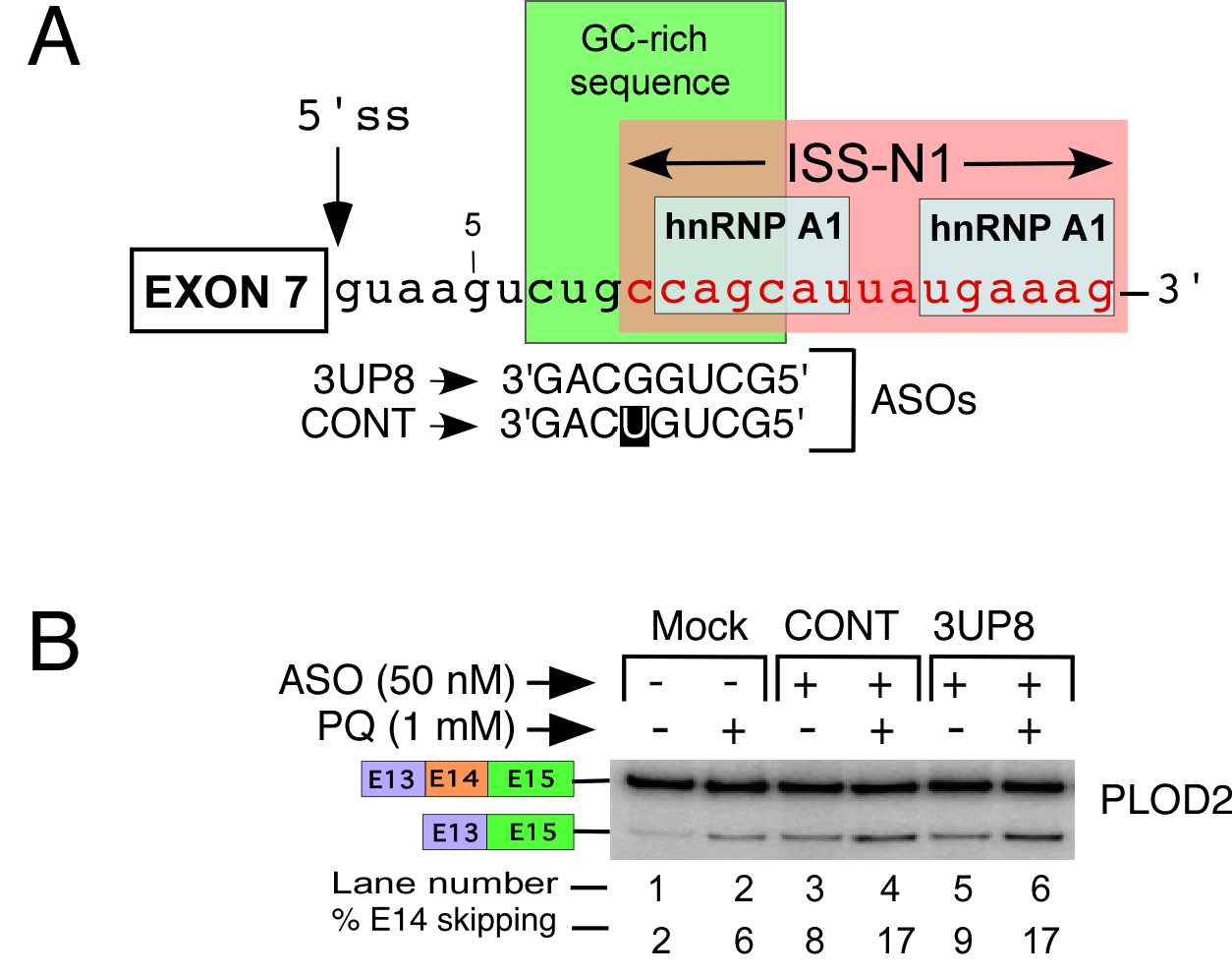

Supplement: Figure S5 — Results showing specificity of 8-mer ASO targeting GC-rich sequence. A, Diagrammatic representation of the ASO target area as described in Figure 7A. B, Splicing pattern of endogenous PLOD2 in SMA patient fibroblasts transfected with different ASOs in the presence (+) or absence (−) of PQ. Transfection, PQ treatment and RNA isolation procedures were the same as described in Figure 7. PLOD2 spliced products were analyzed by RT-PCR. RNA was converted to cDNA using an oligo(dT)12–18 primer. PLOD2 transcripts were amplified using E13-PLOD2 and E15-PLOD2 primers that anneal to exons 13 and 15, respectively. Primer sequences are given in Table S1. (DOCX) [file pone.0049595.s005.docx]
