# Supplementary material for: A Multi-Exon-Skipping Detection Assay Reveals Surprising Diversity of Splice Isoforms of Spinal Muscular Atrophy Genes
Source: PLoS One. 2012 Nov 19;7(11):e49595. doi: 10.1371/journal.pone.0049595 (PMC3501452; doi:10.1371/journal.pone.0049595)
Supplement: Table S1 — List of primers used in this study. (DOCX) [file pone.0049595.s006.docx]

**Table S1**

| No. | Name | Type | Sequence (5' to 3') | Annealing site |
| --- | --- | --- | --- | --- |
| **Primers for PCR** | | | | |
| 1 | P2 | Reverse | *GCATGCAAG*CTTCCTTTTTTCTTTCCCAACAC | E8 |
| 2 | P1 | Forward | CGACTCACTATAGGCTAGCC | pCI-bb |
| 3 | P2-2 | Reverse | CTTCCTTTTTTCTTTCCCAACAC | E8, MESDA |
| 4 | P25 | Reverse | *CTCGAAGCGGCCG*CAGCTCATAAAATTACCA | E8 |
| 5 | P26 | Reverse | GTACAATGAACAGCCATGTC | E8 |
| 6 | P31 | Forward | CATGAGTGGCTATCATACTG | E6 |
| 7 | P42 | Forward | *GCTAGCAAGCTT*ATAATTCCCCCACCACCTCCC | E6 |
| 8 | P43 | Reverse | *GGCTGGATCC*TGTCATTTAGTGCTGCTCTATG | E8 |
| 9 | P45 | Forward | ACGCGTGTGGCCTCGAACACC | pTK-bb |
| 10 | N-24 | Forward | CCAGATTCTCTTGATGATGCTGATGCTTTGGG | E6 |
| 11 | 5'SMN-pro-BglII | Forward | *GAATCTCGAGAT*CCCGGGCCCGCGGGTGCG | SMN promoter |
| 12 | 3'SMN-pro-XhoI | Reverse | *CTGGAGATCT*GGTACCACAAGCCCATACCAC | SMN promoter |
| 13 | 3'E8-Dde | Reverse | CTACAACACCCTTCTCACAGCTC | E8, MESDA |
| 14 | 3'Jun E8/bb | Reverse | TATCATGTCTGCTCGAAGCGG | pCI bb |
| 15 | 5'hSMN-E2b | Forward | GAATACTGCAGCTTCCTTACAACAG | E2b, MESDA |
| 16 | E13-PLOD2 | Forward | CCGATCAGAGATGAATGAAAGGAAC | E13 |
| 17 | E15-PLOD2 | Reverse | TGCCAGAGGTCATTGTTATAATGGG | E15 |
| **Primers for qPCR** | | | | |
| 18 | 5'E6 | Forward | CCTCCCATATGTCCAGATTCTCTTGA | E6 |
| 19 | 3'E6+7 | Reverse | TCTTTTTGATTTTGTCTGAAACC**CATA** | Jxn E6/E7 |
| 20 | 3'E6+8 | Reverse | TGCTCTATGCCAGCATTTC**CATAT** | Jxn E6/E8 |
| 21 | 5'E5+6 | Forward | TTCCTTCTGGACCACCA**ATAA** | Jxn E5/E6 |
| 22 | 5'E4+6 | Forward | AGACTGGGACCAGGAAA**GATAA** | Jxn E4/E6 |
| 23 | 3'E6 | Reverse | ATAGCCAGTATGATAGCCACTCAT | E6 |
| 24 | 5'E3+4 | Forward | AGAACAAAATGCTCAAGAG**AATGA** | Jxn E3/E4 |
| 25 | 5'E2b+4 | Forward | TGCAGCTTCCTTACAACAG**AATGA** | Jxn E2b/E4 |
| 26 | 3'E4 | Reverse | CTTTCCTGGTCCCAGTCTTG | E4 |
| 27 | 5'E1 | Forward | CGCGGGTTTGCTATGGCGAT | E1 |
| 28 | 3'E1+2a | Reverse | CAGAATCATCGCT**CTGGCCTGT** | Jxn E1/E2a |
| 29 | 5'GAPDH | Forward | AACAGCGACACCCACTCCTC |  |
| 30 | 3'GAPDH | Reverse | CATACCAGGAAATGAGCTTGACAA |  |

**Abbreviations**, MESDA, multi-exon-skipping detection assay; E, Exon; Jxn, Junction; bb, backbone. Extra sequences for cloning are shown in Italic; junction sites are in bold.
