# Supplementary material for: A Multi-Exon-Skipping Detection Assay Reveals Surprising Diversity of Splice Isoforms of Spinal Muscular Atrophy Genes
Source: PLoS One. 2012 Nov 19;7(11):e49595. doi: 10.1371/journal.pone.0049595 (PMC3501452; doi:10.1371/journal.pone.0049595)
Supplement: Materials and Methods S1 — Detailed information on methods used in this study. (DOCX) [file pone.0049595.s007.docx]

**Materials and Methods S1**

**Identification of *SMN* splice isoforms.** Endogenous *SMN* spliced products were amplified using Taq DNA polymerase and either primers 5'hSMN-E2b and P2-2 or 5'hSMN-E2b and 3'E8-Dde. cDNA produced with oligo(dT)_12-18_ primer was used as a template. PCR amplification was performed for 30 to 35 cycles. PCR products were resolved on a 6% native polyacrylamide gel and visualized by ethidium bromide staining. Bands corresponding to different splice isoforms were excised from a gel, and the “crush and soak” method^48^ followed by ethanol precipitation was used to recover PCR products. The recovered products were subsequently cloned in a pGEM-T easy vector following the manufacturer’s recommendations (Promega). Recombinant clones were identified by white/blue colony screening on indicator plates. Eight or more clones were randomly selected for each splice variant. Clones were purified using QIAprep Spin Miniprep Kit (Qiagen) and sequenced. *SMN1* and *SMN2* splice isoforms were distinguished based on the absence and presence of DdeI restriction site in exon 8, respectively, and/or by the nucleotide identity at position +6 in exon 7.

**Quantitative real-time PCR (qPCR).** Total RNA from PQ treated and untreated SH-SY5Y cells was isolated using Trizol reagent (Life Technologies) following the manufacturer’s recommendations. To generate cDNA, reverse transcription was carried out using a SuperScript III reaction kit (Life Technologies) with oligo(dT)_12-18_ (Life Technologies) as a primer. 1 μg of total RNA was used per 10 μl of Reverse transcriptase (RT) reaction. All qPCR reactions were performed in triplicates on a Stratagene MxPro 3005P QPCR System. In amplification step 3 μl of 1:20 diluted RT reaction were used as a template in 20 μl of qPCR reaction. Reaction also contained 300 nM of each primer and 1X FastStart Universal SYBR Green Master (ROX) mix (Roche Applied Science). For negative control, nuclease-free water was used instead of cDNA. The primer sets for each assay were designed to anneal to specific exon and exon-exon junctions to distinguish different splice isoforms and avoid amplification of genomic DNA. The amplicon sizes were between 85 and 172 bp. The following primers were used for amplification of *SMN* splice isoforms: primers 5'E1 and 3'E1+2a for all *SMN* isoforms (total *SMN*); primers 5'E6 and 6'E6+7 for exon 7 included transcripts (7+); primers 5'E6 and 6'E6+8 for exon 7 skipped transcripts (Δ7); primers 5'E5+6 and 3'E6 for exon 5 included transcripts (5+); primers 5'E4+6 and 3'E6 for exon 5 skipped transcripts (Δ5); primers 5'E3+4 and 3'E4 for exon 3 included transcripts (3+); primers 5'E2b+4 and 3'E4 for exon 3 skipped transcripts (Δ3). For normalization, we first used Glyceraldehyde 3-Phosphate Dehydrogenase (GAPDH) transcripts that we amplified using primers 5'GAPDH and 3'GAPDH (reference gene). Subsequently all splice variants (from PQ treated and untreated samples) were normalized by total *SMN* from untreated cells. All primer sequences used in qPCR are given in Supplemental Table S1.

**Statistical analysis.** Relative quantities with standard deviations were calculated by using delta-delta CT method by Excel (Microsoft Office 2011 edition). Data were expressed as + standard deviation. Statistical analyses were performed using the unpaired Student’s t-test. Unless otherwise mentioned, P values were two-tailed and the level of statistical significance was set at P<0.05.
